# Mechanically Triggered DNA Nanovehicles for Targeted Dual‐Drug Cancer Therapy

**DOI:** 10.1002/advs.75286

**Published:** 2026-04-15

**Authors:** Murali Mohana Rao Singuru, Priyanka Bhattacharyya, Mingxu You

**Affiliations:** ^1^ Department of Chemistry University of Massachusetts Amherst Amherst Massachusetts USA; ^2^ Molecular and Cellular Biology Graduate Program University of Massachusetts Amherst Amherst Massachusetts USA

**Keywords:** DNA nanovehicle, dual‐drug delivery, integrin‐mediated forces, intercellular forces, targeted cancer therapy

## Abstract

Stimuli‐responsive dual‐drug delivery systems enable precise therapeutic control by co‐releasing multiple agents, enhancing synergy while reducing dosage and side effects. Mechanical dysregulation is a hallmark of various diseases, yet force‐responsive platforms remain rare. Here, we introduce a DNA‐based mechanical nanovehicle that releases two anticancer drugs—TMPyP4 and doxorubicin—in response to tensile forces generated by integrin receptors at cell–cell junctions. The cholesterol‐modified DNA constructs anchor to the cell membrane and undergoes force‐induced structural changes, triggering rapid drug release under defined mechanical conditions. Our studies demonstrated selective activation in HeLa and MCF‐7 cancer cells, achieving potent cytotoxicity while minimizing off‐target effects in low‐tension HEK293T cells. This modular platform integrates mechanosensing, real‐time force visualization, and targeted therapy, establishing a new class of mechanoresponsive dual‐drug delivery systems for safer and more effective cancer treatments.

## Graphical abstract

A mechanically triggered DNA nanovehicle is introduced that co‐releases two anticancer drugs in response to integrin‐mediated cellular forces. Cholesterol‐anchored DNA assemblies undergo force‐induced unfolding at cell–cell junctions, enabling selective, localized dual‐drug activation in cancer cells while minimizing off‐target toxicity in low‐tension cells.

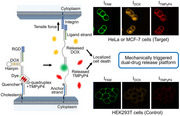

## 1 Introduction

Stimuli‐responsive dual‐drug delivery systems represent a major advancement in therapeutic design, offering improved efficacy with reduced side effects. Unlike conventional therapies that distribute drugs systematically, these platforms release their payloads selectively at disease sites such as tumors or inflamed tissues. This targeted approach enables personalized “cocktail therapies” that combine modalities like chemotherapy, gene therapy, and radiotherapy to match individual disease profiles [1, 2, 3]. To achieve this precision, delivery systems leverage mechanisms such as ligand–receptor interactions, pH‐ or temperature‐sensitive carriers, and enzyme‐, redox‐, or biomolecule‐responsive materials [4, 5, 6, 7, 8]. Once localized, these carriers release therapeutic agents in response to specific biochemical or physical triggers. Co‐delivery of multiple drugs further allows synchronized release and synergistic effects, minimizing harm to healthy tissues while enabling potent, controlled treatment.

Compared to other stimulus‐responsive delivery platforms, although mechanical force‐activated payload release is an emerging and promising strategy, enabled by both external forces and endogenous mechanical cues [9, 10, 11, 12], drug delivery systems capable of responding to mechanical forces with cellular‐level precision remain uncommon [13, 14, 15, 16]. Yet these cellular forces are essential for processes such as adhesion, migration, differentiation, proliferation, and signaling. Mechanical cues are transmitted through membrane‐bound receptors and mechanosensitive proteins, including integrins, cadherins, Piezo1/2 channels, Notch receptors, and immune cell receptors. Many of these mechanotransducers serve as biomarkers and therapeutic targets in diseases ranging from cancer and fibrosis to autoimmune disorders and diabetes [17, 18, 19, 20, 21]. Harnessing cell‐generated mechanical signals offers a powerful strategy for precision drug delivery. Mechano‐responsive systems can be designed to release therapeutic cargo only in cells exhibiting specific force signatures, ensuring activation at the intended site [13, 14, 15, 16]. As mechanotransduction research advances, force‐responsive delivery holds strong potential for next‐generation platforms with cellular‐level accuracy and control.

We recently developed a targeted drug delivery platform that activates on demand in response to molecular forces at cell–cell junctions [16]. This system uses a cholesterol‐modified DNA hairpin nanostructure that spontaneously anchors to live‐cell membranes and releases drug molecules when exposed to receptor‐generated tensile forces in situ. We demonstrated that doxorubicin (DOX), a powerful anticancer drug capable of intercalating into DNA duplexes, can be loaded within the hairpin stem. When intercellular tensile forces exceed the unfolding threshold, the hairpin opens, rapidly releasing DOX for uptake by adjacent cells, resulting in the first intercellular molecular force‐triggered targeted cell apoptosis. This approach enables cancer cell‐specific drug activation and offers a potentially safer therapeutic strategy.

To extend this concept to dual‐drug delivery beyond DNA hairpins, we engineered in this study a multifunctional DNA nanostructure for “cocktail therapy” that responds to mechanical forces generated by cell surface receptors. This nanodevice combines a cholesterol‐modified G‐quadruplex and a DNA hairpin, each carrying a distinct therapeutic agent: TMPyP4 (a telomerase inhibitor) and DOX. Both drugs remain stably bound until receptor–ligand interactions generate forces exceeding the unfolding threshold (F_1/2_, see Supporting Information), triggering structural opening and rapid, localized release. Once released, TMPyP4 stabilizes G‐quadruplex structures, inhibiting telomere maintenance and suppressing cancer cell proliferation, while DOX intercalates into DNA and inhibits topoisomerase II, inducing double‐strand breaks and halting cell division. Although TMPyP4 and DOX exhibit strong and synergistic anticancer effects [22, 23, 24, 25], clinical use is limited by poor selectivity and systemic toxicity. Embedding both agents in a mechano‐responsive DNA scaffold enables force‐triggered activation specifically within cancer cells, offering a safer and more precise approach to combination therapy.

## 2 Results and Discussion

### 2.1 Design and Characterization of G‐Quadruplex DNA Mechanical Nanovehicle (GDMV)

To demonstrate feasibility, we focused on intercellular tensile forces generated by the integrin α_v_β_3_ receptor, which binds Arg‐Gly‐Asp (RGD) motifs. This receptor is highly enriched on tumor‐associated endothelial cells, making it an attractive therapeutic target [26, 27]. Beyond its selective expression, α_v_β_3_ plays a critical role in tumor angiogenesis, metastasis, and immune cell trafficking within the tumor microenvironment. When engaged with peptide ligands, α_v_β_3_ transmits piconewton‐scale forces—typically ∼10–50 pN, depending on cell type and tissue [28]. Exploiting these mechanically transmitted forces enables the design of dual‐drug delivery systems that activate specifically in response to α_v_β_3_ activity, paving the way for more precise and effective cancer treatments.

**FIGURE 1:**
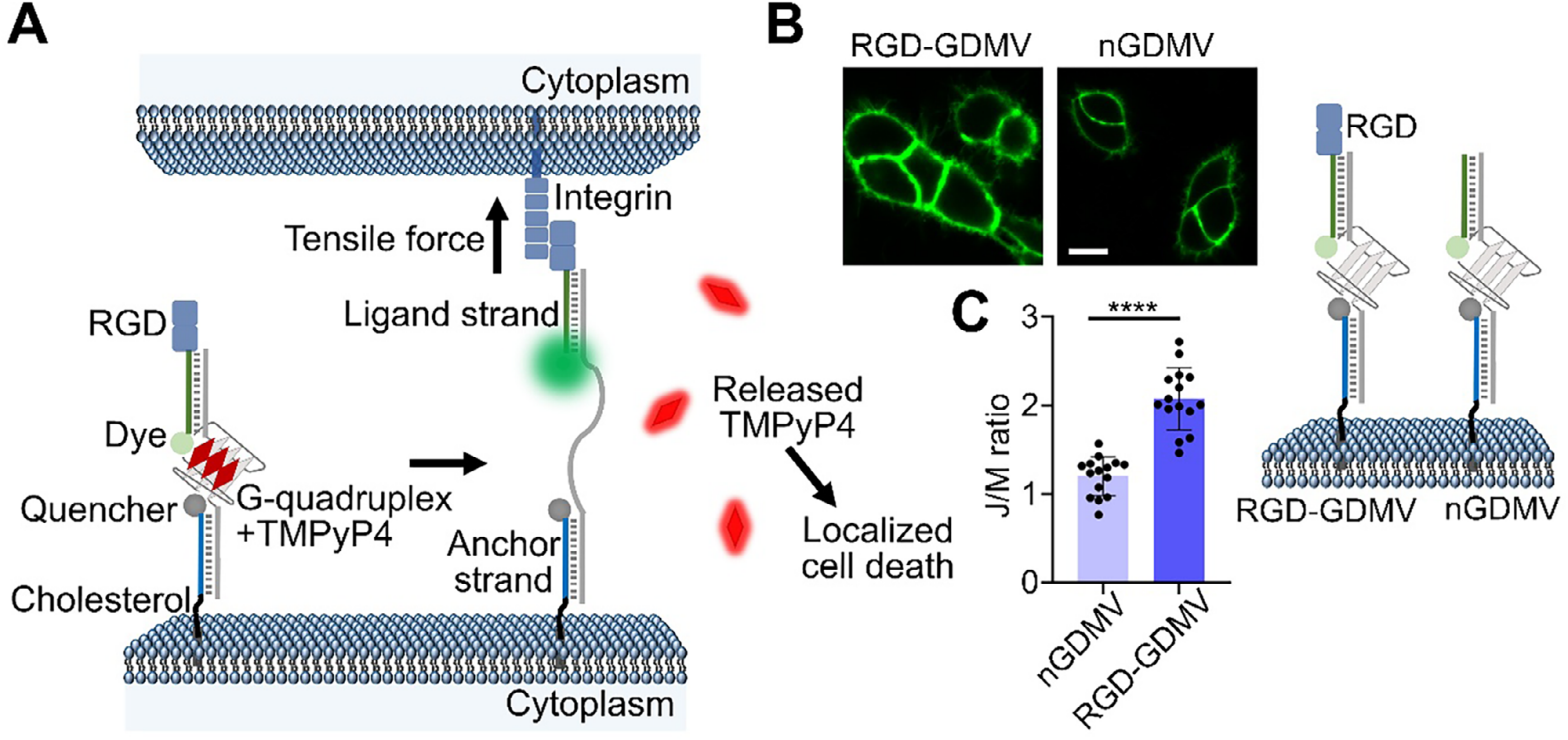
Mechanically triggered activation of G‐quadruplex DNA nanovehicles at cell−cell junctions. (A) Schematic of a G‐quadruplex‐based DNA mechanical nanovehicle at cell−cell junctions. Tensile forces unfold the G‐quadruplex, separating the fluorophore−quencher pair and releasing the intercalated telomerase inhibitor (TMPyP4) to induce targeted apoptosis. (B) Representative confocal images (FAM channel) of HeLa cells incubated for 30 min with 500 nm RGD‐GDMV or nGDMV. Samples were rinsed twice with HEPES before imaging. Scale bar: 20 µm. Schematics of RGD‐GDMV and nGDMV are shown. (C) Quantification of J/M FAM fluorescence ratios across 15 HeLa cell−cell contacts. Shown are the mean and standard deviation (SD) values in each case. Statistical significance was determined using an unpaired two‐tailed Student's *t*‐test (^****^ *p* < 0.0001).

We first designed a DNA nanostructure that functions as both a mechanical force sensor and a drug delivery vehicle. The construct incorporates a cholesterol‐modified human telomere hybrid‐1 G‐quadruplex functionalized with a cyclic Arg‐Gly‐Asp‐D‐Phe‐Lys (cRGDfk, abbreviated as RGD) peptide, which binds integrin receptors such as α_v_β_3_, α_5_β_1_, and α_IIb_β_3_—key mediators of adhesion, migration, and mechanotransduction on cancer cell membranes [29]. As a proof of concept, we assembled a G‐quadruplex‐based DNA mechanical nanovehicle (**GDMV**) from three synthetic oligonucleotides forming a stable architecture via two 21‐base‐pair handles (Figure 1A). The ligand strand carries the RGD peptide and a FAM fluorophore, while the anchor strand includes a cholesterol moiety and an Epoch Eclipse quencher (Table S1), enabling membrane insertion and fluorescence reporting. When intact, the fluorophore and quencher remain close, suppressing fluorescence; however, tensile forces exceeding ∼20 pN unfold the G‐quadruplex [30], separating the pair and producing a detectable fluorescence signal.

Formation of G‐quadruplex within GDMV was confirmed using thioflavin T, which showed enhanced fluorescence upon binding (Figure S1A). Small molecules such as thioflavin T and TMPyP4—a cationic porphyrin derivative and telomerase inhibitor—associate strongly with the G‐rich quadruplex region through end‐stacking and intercalation. To quantify TMPyP4 loading, we characterized its absorbance and fluorescence in the presence of the G‐quadruplex. Incubation of 1 µm G‐quadruplex strand with 5 µm TMPyP4 caused a ∼50% decrease in absorbance and a bathochromic shift from ∼423 to ∼440 nm (Figure S1B). Correspondingly, TMPyP4 fluorescence decreased by ∼55% (Figure S1C), and a redshift in the emission spectrum was also observed, reflecting microenvironmental changes attributed to enhanced *π*–*π* stacking and increased rigidity within the G‐quadruplex framework. Measurement of residual TMPyP4 in the supernatant confirmed efficient binding of ∼3 µm TMPyP4, demonstrating strong drug‐loading capacity in the GDMV (Figure S1C,D).

To prepare the ligand‐functionalized nanostructure, we conjugated the thiolated DNA ligand strand with the RGD peptide using the heterobifunctional cross‐linker sulfo‐SMCC. Conjugation was confirmed by polyacrylamide gel electrophoresis, which showed the expected migration shift (Figure S2A). The three DNA components—RGD‐modified ligand strand, cholesterol‐modified anchor strand, and G‐quadruplex strand—were then assembled into the complete RGD‐functionalized GDMV (RGD‐GDMV), with agarose gel electrophoresis verifying proper assembly (Figure S2B). The quenching efficiency of FAM by the adjacent Epoch Eclipse quencher within the RGD‐GDMV was approximately 70% (Figure S2C).

### 2.2 Integrin‐Mediated Mechanical Activation of GDMV at Cell–Cell Junctions

The functional performance of RGD‐GDMV was then evaluated in HeLa cells, a human epithelial cancer cell line with high surface expression of integrin α_v_β_3_, a key mediator of cell adhesion and mechanotransduction. HeLa cells were incubated with 500 nm RGD‐GDMV for 30 min without TMPyP4 loading, and a control nanostructure lacking the RGD peptide (nGDMV) was also tested. Confocal imaging confirmed efficient membrane incorporation for both constructs, consistent with cholesterol‐mediated anchoring. However, RGD‐GDMV produced strong, localized fluorescence at cell–cell junctions, whereas nGDMV showed substantially weaker signals (Figure 1B). To quantify this, we calculated the junction‐to‐membrane (J/M) fluorescence ratio, defined as fluorescence intensity at junctions divided by the average intensity across other membrane regions. RGD‐GDMV exhibited a J/M ratio of ∼2.1, indicating nearly twofold enhancement at junctions, while nGDMV remained near 1.2 (Figure 1C). These results demonstrate that RGD‐GDMV effectively reports integrin‐mediated mechanical forces on the RGD ligands at cell–cell contacts, whereas the control lacks force responsiveness.

**FIGURE 2:**
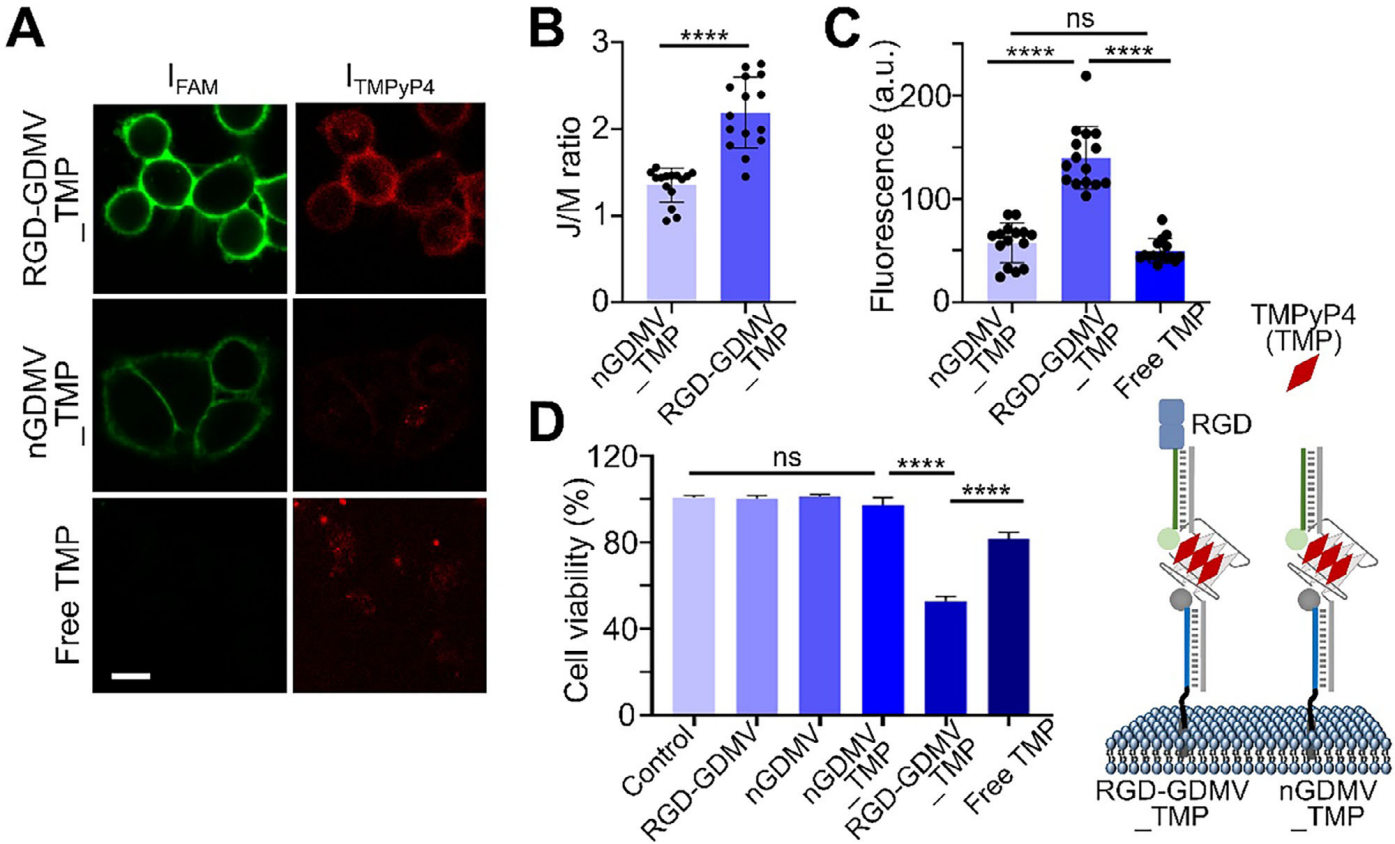
Tensile force–triggered TMPyP4 release at cell–cell junctions. (A) Representative confocal images of HeLa cells after a 4‐h incubation with 3.0 µm free TMPyP4 or 3 µm TMPyP4‐loaded 1.0 µm RGD‐GDMV/nGDMV. Samples were rinsed twice with HEPES before imaging. Scale bar: 20 µm. (B) Quantification of J/M FAM fluorescence ratios (mean ± SD) from 15 junctional cells. Statistical significance was determined by unpaired two‐tailed Student's *t*‐test (^****^ *p* < 0.0001). (C) Quantification of cellular TMPyP4 fluorescence (mean ± SD) from at least 15 junctional cells. Significance was assessed by one‐way ANOVA (ns = not significant, ^****^ *p* < 0.0001). (D) XTT assay evaluating cytotoxicity after a 4‐h incubation with 3.0 µm free TMPyP4, 1.0 µm RGD‐GDMV/nGDMV, or 3 µm TMPyP4‐loaded 1.0 µm RGD‐GDMV/nGDMV. Schematics of free TMPyP4 and TMPyP4‐loaded RGD‐GDMV and nGDMV are shown. Untreated cells served as controls. Data represents mean ± SD from three biological replicates. Significance was determined by one‐way ANOVA (ns = not significant, ^****^ *p* < 0.0001).

We next evaluated whether RGD‐GDMV retained its mechano‐responsive behavior after TMPyP4 loading. TMPyP4 and FAM can be selectively excited at 641 and 488 nm, respectively, allowing clear channel separation. To confirm this, HeLa cells were incubated with 1 µm RGD‐GDMV or 3 µm free TMPyP4, and fluorescence imaging was performed under both excitation conditions. After optimizing laser power and background thresholds, the two reporters produced distinct, non‐overlapping emission patterns. Using this configuration, we measured intracellular FAM and TMPyP4 fluorescence 4 h after treatment with TMPyP4‐loaded RGD‐GDMV (RGD‐GDMV_TMP) or the non‐targeting control (nGDMV_TMP). As expected for a force‐activated construct, RGD‐GDMV_TMP showed a J/M fluorescence ratio of ∼2.2 on the FAM channel, compared to ∼1.3 for nGDMV_TMP (Figure 2A,B). Intracellular TMPyP4 levels were also ∼1.5‐fold higher in the RGD‐GDMV_TMP group, confirming that mechanical tension effectively triggered TMPyP4 release inside cancer cells (Figure 2C).

After confirming the tension‐dependent release mechanism, we tested whether mechanically triggered TMPyP4 release could be used for targeted cancer therapy. XTT cell viability and proliferation assays showed that nGDMV_TMP caused minimal cytotoxicity after 4 h, indicating negligible drug release without mechanical activation. In contrast, RGD‐GDMV_TMP induced rapid and significant cell death within the same period. Notably, its therapeutic effect surpassed that of 3 µm free TMPyP4 (Figure 2D), likely due to elevated local drug concentration at the membrane upon force‐triggered release.

As an additional control, we developed a neuregulin‐1‐modified DNA nanovehicle (NRG1‐GDMV) targeting ErbB3 (HER3) and ErbB4 (HER4) receptors. Given the comparable expression levels of HER3 and integrin α_v_β_3_ on MCF‐7 cells, as well as their similarly high ligand–receptor affinities (low nanomolar K_D_) [31, 32], NRG1‐GDMV was used to validate the role of intercellular forces in TMPyP4 release. As shown in Figure S3, both cytoplasm‐to‐membrane TMPyP4 fluorescence ratios and XTT cytotoxicity assays indicate that 3 µm TMPyP4‐loaded 1.0 µm RGD‐GDMV exhibited significantly greater drug release at MCF‐7 cell junctions. These results support the conclusion that drug release is governed by intercellular mechanical forces rather than receptor binding alone. Collectively, these findings demonstrate that RGD‐GDMV retains its force‐responsive capability after TMPyP4 loading, effectively translating integrin‐mediated mechanical cues into drug release and providing a powerful strategy for targeted chemotherapeutic delivery in cancer cells.

### 2.3 Engineering and Characterization of Dual‐Drug Mechanical Nanovehicle (DDMV)

**FIGURE 3:**
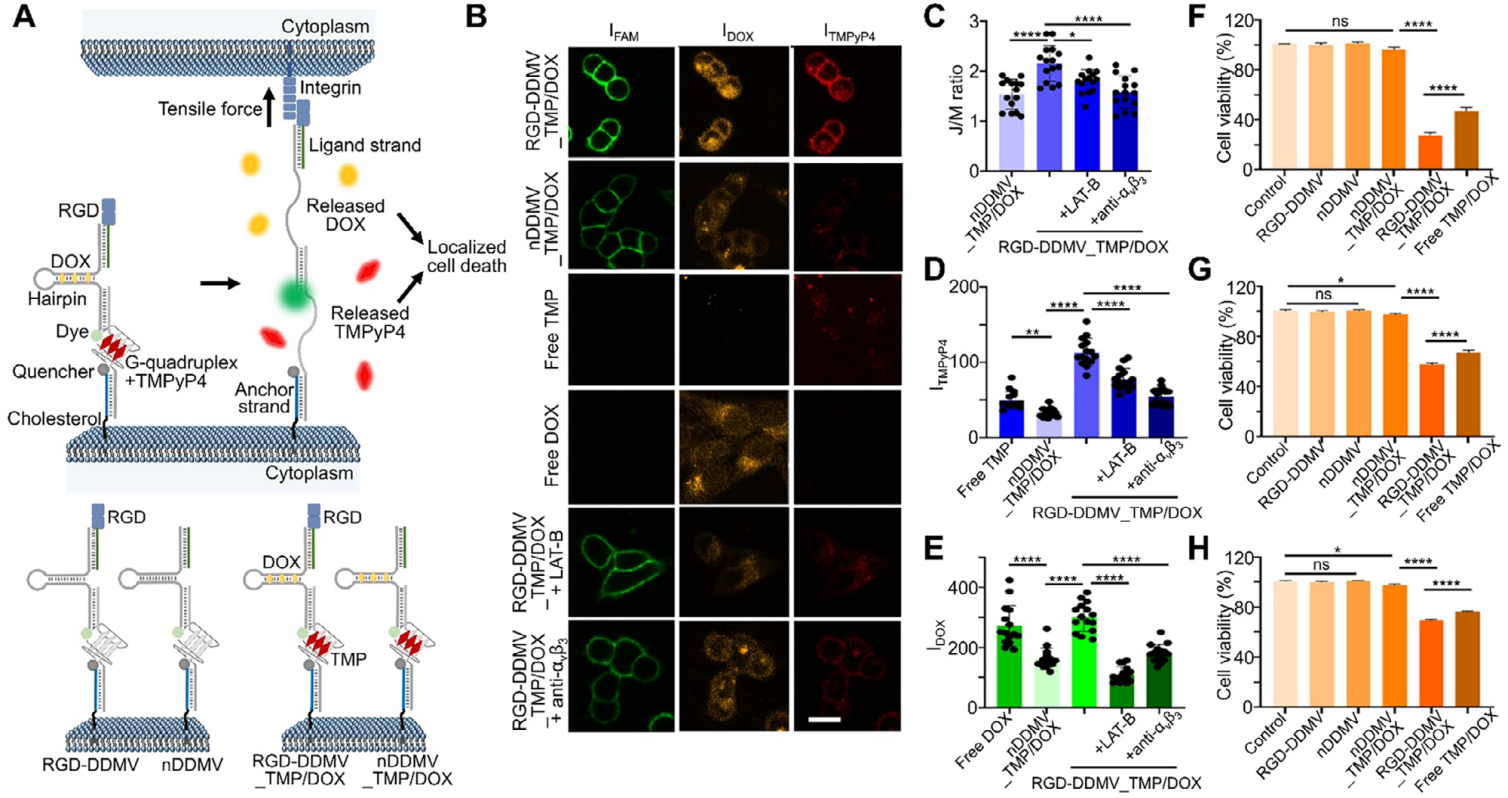
Mechanically triggered dual‐drug release at HeLa cell junctions. (A) Schematic of a DNA‐based mechanical nanovehicle for dual‐drug delivery. Tensile forces at cell–cell junctions unfold the G‐quadruplex and hairpin structure, separating the fluorophore–quencher pair and releasing both TMPyP4 and DOX to induce targeted apoptosis. Schematics of RGD‐DDMV, nDDMV, and TMPyP4/DOX‐loaded nanovehicles are shown. (B) Representative fluorescence images of HeLa cells after a 4‐h incubation with 3.0 µm free TMPyP4/DOX or 3 µm TMPyP4/DOX‐loaded 1.0 µm RGD‐DDMV/nDDMV. Samples were rinsed twice with HEPES before imaging. For force‐inhibition studies, cells were pretreated with 1.0 µm latrunculin B (20 min) or 10 µg/mL anti‐α_v_β_3_ antibody (30 min) before exposure to TMPyP4/DOX‐loaded RGD‐DDMV. Scale bar: 20 µm. (C) Quantification of J/M FAM fluorescence ratios (mean ± SD) from 15 junctional cells in each case. Significance was determined by one‐way ANOVA (ns = not significant, ^***^ *p* < 0.001, ^****^ *p* < 0.0001). (D) Cellular TMPyP4 fluorescence (mean ± SD) as measured from 15 junctional cells. Statistical significance was assessed by one‐way ANOVA (ns = not significant, ^*^ *p* < 0.05, ^****^ *p* < 0.0001). (E) Cellular DOX fluorescence (mean ± SD) from 15 junctional cells. Significance was determined by one‐way ANOVA (ns = not significant, ^****^ *p* < 0.0001). (F) HeLa cells were treated for 4 h with either 3.0 µm free TMPyP4/DOX, 3 µm TMPyP4/DOX‐loaded 1.0 µm RGD‐DDMV/nDDMV, or 1.0 µm RGD‐DDMV/nDDMV alone. Untreated cells served as controls. Shown are mean ± SD from three biological replicates. One‐way ANOVA (ns = not significant, ^****^ *p* < 0.0001). (G) Treatment with 1.5 µm free TMPyP4/DOX, ∼1.5 µm TMPyP4/DOX‐loaded 0.5 µm RGD‐DDMV/nDDMV, or 0.5 µm RGD‐DDMV/nDDMV alone. Mean ± SD (*n* = 3). One‐way ANOVA (ns = not significant, ^*^ *p* < 0.05, ^****^p < 0.0001). (H) Treatment with 0.75 µm free TMPyP4/DOX, ∼0.7 µm TMPyP4/DOX‐loaded 0.25 µm RGD‐DDMV/nDDMV, or 0.25 µm RGD‐DDMV/nDDMV alone. Mean ± SD (*n* = 3). One‐way ANOVA (ns = not significant, ^*^ *p* < 0.05, ^****^ *p* < 0.0001).

To further enable dual‐drug release in response to molecular tension at cell–cell junctions, we conjugated a DNA hairpin strand to the GDMV, creating a four‐stranded, force‐responsive framework assembled via three 21‐base‐pair handles, termed the dual‐drug responsive DNA mechanical nanovehicle (**DDMV**) (Figure 3A). In this design, TMPyP4 is loaded within the G‐quadruplex elements, while DOX intercalates into the DNA hairpins, with each component engineered to unfold at distinct tension thresholds (see Supporting Information). A FAM/Epoch Eclipse fluorophore–quencher pair was incorporated for activation reporting, yielding a quenching efficiency of ∼76% (Figure S2D).

Using this platform, we first tested whether RGD‐functionalized DDMVs could sense intercellular tension and activate their structural switch. HeLa cells were incubated with 500 nm RGD‐DDMV or a non‐targeting control lacking the RGD peptide (nDDMV). Both constructs are associated with the plasma membrane, but strong junctional fluorescence was observed only for RGD‐DDMV, indicating integrin‐mediated force activation (Figure S4A). Quantification showed a J/M ratio of ∼2.1 for RGD‐DDMV compared to ∼1.4 for nDDMV, validating RGD‐dependent mechanosensing (Figure S4B). We next examined the time‐dependent membrane association and internalization of nDDMV over 4–24 h. As shown in Figure S4C,D, intracellular fluorescence progressively increased, accompanied by a corresponding decrease in membrane‐associated signals. Notably, residual membrane fluorescence remained detectable after 24 h.

### 2.4 Force‐Triggered Dual‐Drug Release in Cancer Cells

We next evaluated whether mechanical responsiveness persisted after dual‐drug loading. TMPyP4, DOX, and FAM were imaged using separate excitation channels (488 nm for FAM, 561 nm for DOX, 641 nm for TMPyP4). Four hours after treatment with 1 µm dual‐loaded RGD‐DDMV_TMP/DOX, strong fluorescence activation was observed (Figure 3B). The FAM J/M ratio for RGD‐DDMV_TMP/DOX was ∼2.2, compared to ∼1.5 for nDDMV_TMP/DOX (Figure 3C). Similarly, intracellular TMPyP4 and DOX fluorescence was ∼2.4‐ and 0.9‐fold higher in cells treated with RGD‐bearing constructs than with nDDMV controls (Figure 3D,E). Single‐drug versions showed similar trends: RGD‐DDMV_TMP and RGD‐DDMV_DOX exhibited substantially greater internalization than their non‐targeting counterparts (Figure S5). These results confirm that RGD‐DDMV maintains mechanosensing capability after drug loading and reliably triggers force‐dependent release.

To examine the role of integrin in RGD‐DDMV activation, we focused on integrin α_v_β_3_, a key mechanotransduction receptor in HeLa cells (Figure 3B). Blocking α_v_β_3_ reduced the FAM J/M ratio of RGD‐DDMV_TMP/DOX from ∼2.2 to ∼1.6, though this remained above the nDDMV control (Figure 3C). Drug fluorescence showed a similar trend: inhibition of α_v_β_3_ lowered TMPyP4 and DOX uptake by ∼52% and 40%, respectively, while nDDMV exhibited an even greater reduction (∼70% and 46%) (Figure 3D,E). Treatment with 1 µm latrunculin B (LAT‐B), which disrupts actin polymerization and blocks integrin‐mediated tension, also eliminated junctional FAM activation and significantly reduced drug uptake (Figure 3B–E). These results confirm that mechanical unfolding of the nanostructure is essential for drug release.

### 2.5 Mechanically Triggered Targeted Dual‐Drug Cancer Therapy

We then evaluated the potential of mechanically triggered TMPyP4 and DOX release for targeted cancer therapy. XTT assays showed that HeLa cells treated with 1 µm RGD‐DDMV_TMP/DOX exhibited rapid and significant cytotoxicity within 4 h (Figure 3F), whereas nDDMV_TMP/DOX caused minimal toxicity, confirming the requirement of mechanical activation in drug release. Remarkably, the therapeutic effect of RGD‐DDMV_TMP/DOX was comparable to or exceeded that of 3 µm free TMPyP4/DOX, likely due to the high local drug concentration achieved during force‐triggered release. Single‐drug constructs again displayed similar trends, with RGD‐functionalized systems consistently outperforming non‐targeting controls (Figure S6A,B).

**FIGURE 4:**
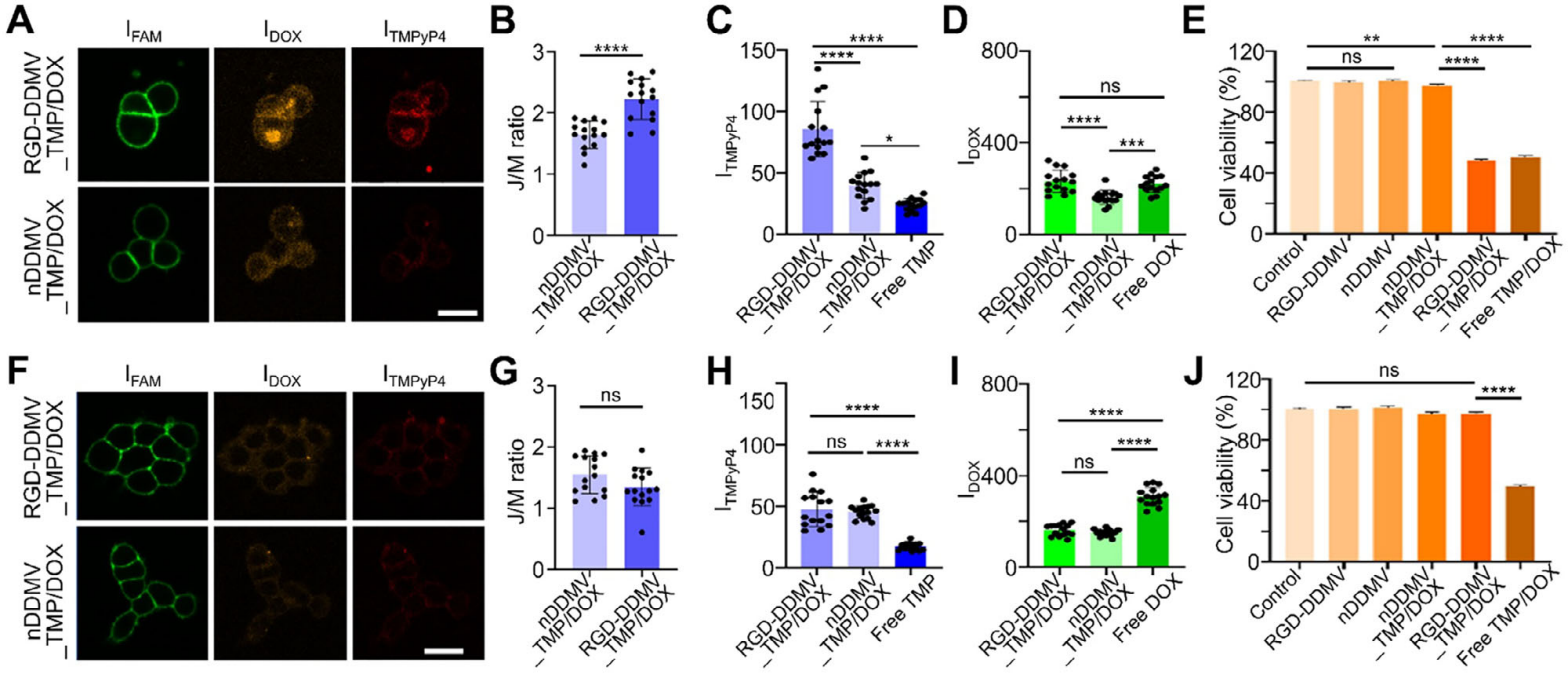
Selective dual‐drug release and cytotoxicity in cancer vs. low‐tension cells. (A) Confocal images of MCF‐7 cells after 4‐hincubation with 1.0 µm RGD‐DDMV_TMP/DOX or nDDMV_TMP/DOX and rinsed twice with HEPES before imaging. Scale bar: 20 µm. (B) Quantification of J/M FAM fluorescence ratios (mean ± SD) from 15 junctional cells in each case. Significance was determined by a two‐tailed Student's *t*‐test (^****^ *p* < 0.0001). (C) Cellular TMPyP4 fluorescence (mean ± SD) as measured from 15 junctional MCF‐7 cells. Statistical significance was assessed by one‐way ANOVA (ns = not significant, ^****^ *p* < 0.0001). (D) Cellular DOX fluorescence (mean ± SD) from 15 junctional cells. Significance was determined by one‐way ANOVA (^*^ *p* < 0.05, ^**^ *p* < 0.01, ^****^ *p* < 0.0001). (E) MCF‐7 cells were treated for 4 h with 3.0 µm TMPyP4/DOX, 3 µm TMPyP4/DOX‐loaded 1.0 µm RGD‐DDMV/nDDMV, or 1.0 µm RGD‐DDMV/nDDMV alone. Untreated cells served as controls. Shown are mean ± SD from three biological replicates. One‐way ANOVA (ns = not significant, ^*^ *p* < 0.05, ^****^ *p* < 0.0001). (F) Confocal images of HEK293T cells after 4‐h incubation with 3 µm TMPyP4/DOX‐loaded 1.0 µm RGD‐DDMV or nDDMV and rinsed twice with HEPES before imaging. Scale bar: 20 µm. (G) Quantification of J/M FAM fluorescence ratios (mean ± SD) from 15 junctional cells in each case. Significance was determined by a two‐tailed Student's *t*‐test (ns = not significant). (H) Cellular TMPyP4 fluorescence (mean ± SD) as measured from 15 junctional HEK293T cells. Statistical significance was assessed by one‐way ANOVA (^*^ *p* < 0.05, ^****^ *p* < 0.0001). (I) Cellular DOX fluorescence (mean ± SD) from 15 junctional cells. Significance was determined by one‐way ANOVA (ns = not significant, ****p < 0.0001). (J) HEK293T cells were treated for 4 h with 3.0 µm TMPyP4/DOX, 3 µm TMPyP4/DOX‐loaded 1.0 µm RGD‐DDMV/nDDMV, or 1.0 µm RGD‐DDMV/nDDMV alone. Untreated cells served as controls. Shown are mean ± SD from three biological replicates. One‐way ANOVA (ns = not significant, ^****^ *p* < 0.0001).

Concentration‐dependent cytotoxicity was also assessed using 0.25 and 0.5 µm RGD‐DDMV_TMP/DOX or nDDMV_TMP/DOX, with 0.75 and 1.5 µm free TMPyP4/DOX as controls (Figure 3G,H; Figure S6C–F). Lower nanodevice concentrations resulted in proportionally reduced cytotoxicity, while nDDMV formulations consistently exhibited minimal cell death. The DNA nanovehicle itself showed no inherent toxicity. To evaluate generalizability, the dual‐drug nanovehicle was applied to MCF‐7 breast cancer cells, which also express α_v_β_3_. These cells displayed robust mechanosensing and drug release responses similar to HeLa cells, confirming the broad applicability of DDMV across integrin‐rich cancer types (Figure 4A–E; Figure S7).

We next examined whether the nanovehicle could minimize off‐target effects in cells with weak intercellular forces. HEK293T cells, which exhibit α_v_β_3_/α_v_β_5_ expression and correspondingly low integrin‐mediated tension, showed only modest FAM activation at junctions (J/M ∼1.4), compared to ∼2.2 in HeLa cells (Figure 4F,G). When loaded with 3 µm TMPyP4/DOX at 1 µm, neither RGD‐DDMV_TMP/DOX nor nDDMV_TMP/DOX, alone or in combination, induced significant cytotoxicity in HEK293T cells (Figure 4H–J; Figure S8). Only a slight reduction was observed with RGD‐DDMV_TMP/DOX, far below the effect of the free drug (Figure 4J). Single‐drug constructs showed similar minimal activation, suggesting that low basal integrin activity can partially—but inefficiently—trigger the device (Figure S8). Further reducing nanodevice concentration or adjusting the hairpin force threshold may help suppress off‐target release.

## 3 Conclusions

In summary, we developed an intercellular force‐responsive DNA nanovehicle for highly selective dual‐drug delivery. By incorporating TMPyP4, a telomerase inhibitor, and DOX, a chemotherapeutic, into cholesterol‐modified DNA assemblies, we achieved precise, force‐triggered release on the membranes of HeLa and MCF‐7 cancer cells. These nanodevices demonstrated strong therapeutic efficacy with minimal off‐target effects in low‐tension HEK293T cells. The modular design allows substitution of the RGD ligand with other targeting motifs, enabling interaction with diverse mechanotransduction‐associated receptors. Beyond TMPyP4 and DOX, additional therapeutics can be integrated via aptamer binding or covalent attachment, further expanding system versatility.

Unlike previous mechanoresponsive DNA nanostructures that require extracellular matrix tethering or external forces [13, 14, 15], our lipid‐modified DDMV anchors directly to cell membranes and responds to native molecular forces in situ. DNA programmability enables precise control of activation thresholds by modifying stem sequences and G‐quadruplex topology, supporting selective targeting based on mechanical phenotypes. While further optimization is needed, such as improving in vivo sensitivity and biostability, this work establishes a proof of concept for identifying and treating diseased cells based on mechanical behavior, even when their biochemical markers remain unchanged.

## 4 Experimental Section

### 4.1 Chemicals and Reagents

Unless stated otherwise, all chemicals were obtained from Sigma or Fisher Scientific and used without further purification. DNA oligonucleotides were synthesized and purified from the W. M. Keck Oligonucleotide Synthesis Facility at Yale School of Medicine and from Integrated DNA Technologies. The cyclic Arg‐Gly‐Asp‐D‐Phe‐Lys (cRGDfk) was purchased from Peptides International. Sulfosuccinimidyl 4‐(N‐maleimidomethyl) cyclohexane‐1‐carboxylate (Sulfo‐SMCC), latrunculin B, tris(2‐carboxyethyl) phosphine (TCEP), cationic 5,10,15,20‐tetra‐(N‐methyl‐4‐pyridyl) porphine (TMPyP4), and the CyQUANT XTT viability assay kit were acquired from Thermo Fisher Scientific. Micro Bio‐Spin 6 columns were supplied by Bio‐Rad Laboratories, and doxorubicin hydrochloride (DOX) was obtained from Advanced ChemBlock. Fluorescence measures utilized a modified 4‐(2‐hydroxyethyl)‐1‐piperazineethanesulfonic acid (HEPES) containing 10 mm MgCl_2_, 2 mm CaCl_2_, and 5 mm KCl at pH 7.2.

### 4.2 G‐Quadruplex DNA Intercalation With TMPyP4

A 1.0 µm solution of human telomeric G‐quadruplex (2HY9) DNA was heated to 75°C for 5 min and then gradually cooled down to 25°C at a rate of 1.3°C/min using a Bio‐Rad PCR thermal cycler. The folded DNA was then incubated with 5.0 µm TMPyP4 for 2 h at room temperature to allow intercalation, then the mixture was centrifuged at 10000 rpm for 10 min at room temperature, and the resulting precipitate (TMPyP4‐loaded G‐quadruplex) was collected for nanovehicle assembly. TMPyP4 loading efficiency was quantified by fluorescence measurements of both the precipitate and the supernatant using a PTI fluorimeter (Horiba, Ltd.), with excitation at 520 nm and emission collected from 600–750 nm. Loading efficiency was determined from a TMPyP4 concentration‐dependent fluorescence calibration curve (Figure S1D) by subtracting the amount of unbound TMPyP4 in the supernatant from the total TMPyP4 added.

### 4.3 Synthesis of RGD‐Modified Ligand Strands

25 µL of a thiolated ligand strand (200 µm) was reduced with 10 µL of 100 mm TCEP in PBS (pH 7.2) for 1 h at room temperature, purified using a Bio‐Spin 6 column (pre‐equilibrated with PBS), and reacted with 5 µL of freshly prepared Sulfo‐SMCC (23 mm) for 30 min at room temperature. After purification using a Bio‐Spin 6 column, 10 µL of cRGDfk peptide (10 mg/mL in PBS, pH 7.2) was added and incubated overnight at 4°C. The final RGD‐conjugated strand was further purified using a Bio‐Spin 6 column and stored at 4°C until further use.

### 4.4 Assembly of TMPyP4‐Loaded G‐Quadruplex Mechanical Nanovehicles

To prepare G‐quadruplex mechanical nanovehicle RGD‐GDMV or nGDMV, G‐quadruplex DNA (1.0 µm), ligand strand (1.0 µm; with or without RGD modification), and anchor strand (1.0 µm) were combined and incubated for 2 h at room temperature. TMPyP4‐loaded GDMV was prepared by pre‐incorporating 1.0 µm G‐quadruplex DNA with 5.0 µm TMPyP4 and calibrated following the above protocol.

### 4.5 Force Threshold Calculation

The mechanical stability of the DNA G‐quadruplex and hairpin was evaluated using the F_1/2_ value, which denotes the force at which 50% of the G‐quadruplexes or hairpins are expected to unfold. The F_1/2_ value of the G‐quadruplex structure used in this study has been determined previously [30]. This F_1/2_ value can be calculated as: F_1/2_ = (ΔG_fold_ + ΔG_stretch_) / Δx [33], where ΔG_fold_ is the unfolding free energy when no force is applied, ΔG_stretch_ is the free energy required to stretch the unfolded G‐quadruplex/hairpin from zero force to F = F_1/2_, and Δx is the extension length or opening distance of the G‐quadruplex/hairpin during unfolding. ΔG_fold_ is determined using nearest neighbour free energy parameters derived from IDT OligoAnalyzer at 37°C, 140.5 mm Na^+^, and 0.4 mm Mg^2+^. ΔG_stretch_ can be determined using the formula: ΔG_stretch_ = (k_B_·T·L_0_/L_p_)·(3x^2^/L_0_^2^ – 2x^3^/L_0_^3^) / [4·(1 – x/L_0_)], where k_B_ is the Boltzmann constant, T is the temperature, L_p_ is the persistence length of single‐stranded DNA (around 1.3 nm), L_0_ is the contour length of single‐stranded DNA (approximately 0.63 nm per nucleotide), and x is the G‐quadruplex/hairpin extension from equilibrium, given by 0.44·(n−1) nm for hairpin, where n is the number of nucleotides in the hairpin. The extension length Δx can be calculated as [0.44·(n−1) – 2] nm for hairpin, with 2 nm subtracted to account for the initial distance between the hairpin termini, considering the diameter of the hairpin stem duplex. The extension length of G‐quadruplexes can be more difficult to calculate, considering their versatile conformations. Using this approach, the F_1/2_ values for the DNA G‐quadruplex and hairpin used in this project were estimated to be approximately 20  and 12 pN, respectively.

### 4.6 Dual‐Drug DNA Mechanical Nanovehicle Preparation

To assemble DDMV_TMP/DOX, 1.0 µm G‐quadruplex DNA and 1.0 µm cholesterol‐tagged anchor strand were annealed by heating at 75°C for 5 min, then gradually cooled to 25°C at a rate of 1.3°C/min using a Bio‐Rad PCR thermal cycler. Afterwards, this complex was incubated with 3.0 µm TMPyP4 for 2 h at room temperature. The mixture was then centrifuged at 10000 rpm for 10 min at room temperature, and the resulting precipitate (TMPyP4‐loaded G‐quadruplex) was collected for nanovehicle assembly. TMPyP4 loading efficiency was quantified by fluorescence measurements of both the precipitate and the supernatant using a PTI fluorimeter (Horiba, Ltd.), with excitation at 520 nm and emission collected from 600–750 nm. Loading efficiency was determined from a TMPyP4 concentration‐dependent fluorescence calibration curve (Figure S1D) by subtracting the amount of unbound TMPyP4 in the supernatant from the total TMPyP4 added. Separately, 1.0 µm fluorophore‐labeled DNA hairpin and 1.0 µm ligand strand (with or without RGD modification) were hybridized for 1 h at room temperature, followed by a 2‐h incubation with 3.0 µm doxorubicin. The mixture was then centrifuged at 10 000 rpm for 10 min at room temperature. The dark red precipitate (DOX‐loaded DNA hairpin) will be collected and used for nanovehicle assembly. DOX loading efficiency was quantified by measuring fluorescence emission at 581 nm (excitation at 470 nm) for both the precipitate and the supernatant using a PTI fluorimeter (Horiba, Ltd.). Loading efficiency was determined from a DOX concentration‐dependent fluorescence calibration curve (Figure S1F) by subtracting the amount of unbound DOX in the supernatant from the total DOX added. Finally, the four‐strand complex was formed after another 1‐h incubation.

### 4.7 Cell Culture

All the cell lines were obtained from ATCC. HeLa and HEK293T cells were cultured in a DMEM medium enriched with 4.5 g/L high glucose, 10% fetal bovine serum (FBS), and 1% penicillin‐streptomycin. MCF‐7 cells were cultured in a DMEM medium supplemented with 10% FBS, L‐glutamine, 110 mg/L sodium pyruvate, and 1% penicillin‐streptomycin. These cells were maintained at 37°C with 5% CO_2_ following standard cell culture protocols, and sub‐cultured and plated at around 50% density when they reached about 80% confluency.

### 4.8 Force‐Triggered Drug Release and Imaging

To visualize force‐triggered drug release, HeLa, HEK293T, or MCF‐7 cells (∼3 × 10^4^) were plated in an 8‐well chambered cover glass (Cellvis LLC) and allowed to adhere overnight. Afterwards, the cells were washed twice with HEPES buffer, incubated with 1.0 µm pre‐assembled DNA nanovehicle for 4 h at room temperature, washed three times with HEPES to remove unbound nanovehicles, and imaged using a Nikon A1SP‐Spectral confocal system with a 40× oil‐immersion objective. Fluorescence signals were collected using the following settings: excitation at 405 nm with emission at 425–475 nm for Hoechst 33342, 488 nm excitation with 500–550 nm emission for 6‐fluorescein, 561 nm excitation with 570–620 nm emission for DOX, and 640 nm excitation with 663–738 nm emission for TMPyP4. Quantitative analysis was performed using NIS‐Elements AR and processed in Origin and GraphPad Prism.

### 4.9 XTT Cytotoxicity Assay

HeLa, HEK293T, or MCF‐7 cells (∼4 × 10^4^/well) were seeded in 96‐well plates and allowed to adhere for 12 h. Cells were then treated with TMPyP4/DOX‐loaded DNA mechanical nanovehicles (0.25–1.0 µm) or equivalent free drugs (0.75–3.0 µm) for 4 h at room temperature. After washing with HEPES, cells were incubated with 100 µL fresh DMEM for 20 h at 37°C with 5% CO_2_. Cell viability was assessed using the CyQUANT XTT assay by adding 70 µL working reagent per well and incubating for 4 h at 37°C. Absorbance at 450 and 660 nm was measured in each well with a BioTek Synergy2 plate reader, and cell viability was calculated relative to the untreated control.

### 4.10 Effect of Force‐Inhibiting Drug and Integrin α_v_β_3_ Inhibition

To assess the role of mechanical forces in drug release, HeLa cells (∼3 × 10^4^) were treated with a force inhibitor drug, latrunculin B (0.1 µm) in a 200 µL mixture of 50% DMEM and 50% HEPES (pH 7.2) and incubated for 20 min at 37°C under 5% CO_2_ prior to measurement. To evaluate integrin α_v_β_3_ involvement, HeLa cells (∼3 × 10^4^) were pretreated with an integrin α_v_β_3_‐specific antibody [23C6] (10 µg/mL) for 30 min, followed by incubation with 1.0 µm RGD‐DDMV_TMP/DOX for 4 h. Excess nanovehicles were removed by rinsing with HEPES, and imaging was performed using a Nikon A1SP‐Spectral confocal microscope.

### 4.11 Statistical Analysis

All experiments were performed with at least three biological replicates. Unless otherwise specified, data are presented as mean ± standard deviation (SD). Statistical analyses were conducted using unpaired two‐tailed Student's *t*‐tests for two‐group comparisons or one‐way ANOVA for comparisons involving more than two groups, with *p* < 0.05 considered statistically significant. Analyses were performed using GraphPad Prism 8 and Origin 8.5.

## Conflicts of Interest

The authors declare no conflicts of interest.

## Supporting information




**Supporting File**: advs75286‐sup‐0001‐SuppMat.docx.

## Data Availability

The data that support the findings of this study are available in the Supporting Information of this article.

## References

[1] M. J. Mitchell , M. M. Billingsley , R. M. Haley , M. E. Wechsler , N. A. Peppas , and R. Langer , “Engineering Precision Nanoparticles for Drug Delivery,” Nature Reviews Drug Discovery 20 (2021): 101–124, 10.1038/s41573-020-0090-8. 33277608 PMC7717100

[2] A. M. Vargason , A. C. Anselmo , and S. Mitragotri , “The Evolution of Commercial Drug Delivery Technologies,” Nature Biomedical Engineering 5 (2021): 951–967, 10.1038/s41551-021-00698-w. 33795852

[3] J. Gong , T. Shi , J. Liu , et al., “Dual‐Drug Codelivery Nano Systems: An Emerging Approach for Overcoming Cancer Multidrug Resistance,” Biomedicine & Pharmacotherapy 161 (2023): 114505, 10.1016/j.biopha.2023.114505. 36921532

[4] W. Zhao , Y. Zhao , Q. Wang , T. Liu , J. Sun , and R. Zhang , “Remote Light‐Responsive Nanocarriers for Controlled Drug Delivery: Advances and Perspectives,” Small 15 (2019): 1903060, 10.1002/smll.201903060. 31599125

[5] C. de Las Heras Alarcon , S. Pennadam , and C. Alexander , “Stimuli Responsive Polymers for Biomedical Applications,” Chemical Society Reviews 34 (2005): 276–285, 10.1039/B406727D. 15726163

[6] S. Mura , J. Nicolas , and P. Couvreur , “Stimuli‐Responsive Nanocarriers for Drug Delivery,” Nature Materials 12 (2013): 991–1003, 10.1038/nmat3776. 24150417

[7] V. P. Torchilin , “Multifunctional, Stimuli‐Sensitive Nanoparticulate Systems for Drug Delivery,” Nature Reviews Drug Discovery 13 (2014): 813–827, 10.1038/nrd4333. 25287120 PMC4489143

[8] J. Majumder and T. Minko , “Multifunctional and Stimuli‐Responsive Nanocarriers for Targeted Therapeutic Delivery,” Expert Opinion on Drug Delivery 18 (2021): 205–227, 10.1080/17425247.2021.1828339. 32969740 PMC7904578

[9] P. Ma , X. Lai , Z. Luo , et al., “Recent Advances in Mechanical Force‐Responsive Drug Delivery Systems,” Nanoscale Advances 4 (2022): 3462–3478, 10.1039/D2NA00420H. 36134346 PMC9400598

[10] Y. Zhang , J. Yu , H. N. Bomba , Y. Zhu , and Z. Gu , “Mechanical Force‐Triggered Drug Delivery,” Chemical Reviews 116 (2016): 12536–12563, 10.1021/acs.chemrev.6b00369. 27680291

[11] J. Wang , J. A. Kaplan , Y. L. Colson , and M. W. Grinstaff , “Mechanoresponsive Materials for Drug Delivery: Harnessing Forces for Controlled Release,” Advanced Drug Delivery Reviews 108 (2017): 68–82, 10.1016/j.addr.2016.11.001. 27856307 PMC5285479

[12] P. Cai , B. Hu , W. R. Leow , et al., “Biomechano‐Interactive Materials and Interfaces,” Biomechano‐Interactive Materials and Interfaces 30 (2018): 1800572. 10.1002/adma.201800572 29882230

[13] A. Stejskalová , N. Oliva , F. J. England , and B. D. Almquist , “Biologically Inspired, Cell‐Selective Release of Aptamer‐Trapped Growth Factors by Traction Forces,” Advanced Materials 31 (2019): 1806380, 10.1002/adma.201806380. PMC6375388 30614086

[14] K. Lei and T. Li , “T Cell Force‐Responsive Delivery of Anticancer Drugs Using Mesoporous Silica Microparticles,” Materials Horizons 7 (2020): 3196–3200, 10.1039/D0MH01285H.

[15] A. Velusamy , R. Sharma , S. A. Rashid , H. Ogasawara , and K. Salaita , “DNA Mechano Capsules for Programmable Piconewton Responsive Drug Delivery,” Nature Communications 15 (2024): 704, 10.1038/s41467-023-44061-w. PMC10808132 38267454

[16] M. M. R. Singuru , M. A. Tabrizi , P. Bhattacharyya , A. A. Ali , and M. You , “Force‐Responsive Delivery of Anticancer Drugs via a DNA Mechanical Nanovehicle,” Nano Letters 25 (2024): 336–342, 10.1021/acs.nanolett.4c05076. 39719379 PMC12046514

[17] C. S. Chen , J. Tan , and J. Tien , “Mechanotransduction at Cell‐Matrix and Cell‐Cell Contacts,” Annual Review of Biomedical Engineering 6 (2004): 275–302, 10.1146/annurev.bioeng.6.040803.140040. 15255771

[18] E. Bazellières , V. Conte , A. Elosegui‐Artola , et al., “Control of Cell–Cell Forces and Collective Cell Dynamics by the Intercellular Adhesome,” Nature Cell Biology 17 (2015): 409–420, 10.1038/ncb3135. 25812522 PMC4886824

[19] P. Friedl and D. Gilmour , “Collective Cell Migration in Morphogenesis, Regeneration and Cancer,” Nature Reviews Molecular Cell Biology 10 (2009): 445–457, 10.1038/nrm2720. 19546857

[20] K. H. Vining and D. J. Mooney , “Mechanical Forces Direct Stem Cell Behaviour in Development and Regeneration,” Nature Reviews Molecular Cell Biology 18 (2017): 728–742, 10.1038/nrm.2017.108. 29115301 PMC5803560

[21] R. Yan , Y. Li , S. Chen , et al., “Mechanotransduction in Shaping Immunity: Pathways, Crosstalk, and Pathophysiological Relevance,” Advanced Science 12 (2025): 12164, 10.1002/advs.202512164. PMC12591155 41020578

[22] A. Romaniuk‐Drapała , E. Totoń , N. Lisiak , M. Idzik , and B. Rubiś , “Telomerase Inhibitors TMPyP4 and BIBR 1532 Show Synergistic Antitumor Activity in Combination With Chemotherapeutic Drugs,” Scientific Reports 15 (2025): 32958, 10.1038/s41598-025-13496-0. 41006335 PMC12475129

[23] A. Mokhtar , T. Mohamed , A. O. Eigza , and M. E. El‐Khouly , “Water‐Soluble Porphyrin‐Mediated Enhanced Photodynamic and Chemotherapy Employing Doxorubicin for Breast Cancer,” Lasers in Medical Science 40 (2025): 241, 10.1007/s10103-025-04466-z. 40407940 PMC12102107

[24] Q. Wang , Z. He , H. Zhu , et al., “Targeting Drug Delivery and Efficient Lysosomal Escape for Chemo‐Photodynamic Cancer Therapy by a Peptide/DNA Nanocomplex,” Journal of Materials Chemistry B 10 (2022): 438–449, 10.1039/D1TB02441H. 34951442

[25] X. Zheng , X. Nie , H. Liu , Y. Fang , Y. Zhao , and L. Xia , “TMPyP4 Promotes Cancer Cell Migration at Low Doses, but Induces Cell Death at High Doses,” Scientific Reports 6 (2016): 26592, 10.1038/srep26592. 27221067 PMC4879555

[26] H. Hamidi and J. Ivaska , “Every Step of the Way: Integrins in Cancer Progression and Metastasis,” Nature Reviews Cancer 18 (2018): 533–548, 10.1038/s41568-018-0038-z. 30002479 PMC6629548

[27] J. A. Nemeth , M. L. Cher , Z. Zhou , C. Mullins , S. Bhagat , and M. Trikha , “Inhibition of αvβ3 Integrin Reduces Angiogenesis, Bone Turnover, and Tumor Cell Proliferation in Experimental Prostate Cancer Bone Metastases,” Clinical & Experimental Metastasis 20 (2003): 413–420, 10.1023/A:1025461507027. 14524530

[28] R. Ma , S. A. Rashid , A. Velusamy , et al., “Molecular Mechanocytometry Using Tension‐Activated Cell Tagging,” Nature Methods 20 (2023): 1666–1671, 10.1038/s41592-023-02030-7. 37798479 PMC11325290

[29] T. G. Kapp , F. Rechenmacher , S. Neubauer , et al., “A Comprehensive Evaluation of the Activity and Selectivity Profile of Ligands for RGD‐Binding Integrins,” Scientific Reports 7 (2017): 39805, 10.1038/srep39805. 28074920 PMC5225454

[30] S. Dhakal , Y. Cui , D. Koirala , et al., “Structural and Mechanical Properties of Individual Human Telomeric G‐Quadruplexes in Molecularly Crowded Solutions,” Nucleic Acids Research 41 (2013): 3915–3923, 10.1093/nar/gkt038. 23396442 PMC3616730

[31] S. Lucie , G. Elisabeth , F. Stéphanie , et al., “Clustering and Internalization of Integrin αvβ3 With a Tetrameric RGD‐Synthetic Peptide,” Molecular Therapy 17 (2009): 837–843, 10.1038/mt.2009.29. 19259068 PMC2760123

[32] K. Kani , E. Park , and R. Landgraf , “The Extracellular Domains of ErbB3 Retain High Ligand Binding Affinity at Endosome pH and in the Locked Conformation,” Biochemistry 44 (2005): 15842–15857, 10.1021/bi0515220. 16313187

[33] M. T. Woodside , W. M. Behnke‐Parks , K. Larizadeh , K. Travers , D. Herschlag , and S. M. Block , “Nanomechanical Measurements of the Sequence‐Dependent Folding Landscapes of Single Nucleic Acid Hairpins,” Proceedings of the National Academy of Sciences 103 (2006): 6190–6195, 10.1073/pnas.0511048103. PMC1458853 16606839

