# Supplementary material for: Mechanically Triggered DNA Nanovehicles for Targeted Dual‐Drug Cancer Therapy
Source: Adv Sci (Weinh). 2026 Apr 15;13(39):e75286. doi: 10.1002/advs.75286 (PMC13335000; doi:10.1002/advs.75286)
Supplement: Supplementary file 1 — Supporting File: advs75286‐sup‐0001‐SuppMat.docx. [file ADVS-13-e75286-s001.docx]

**Supporting Information:**

**Mechanically Triggered DNA Nanovehicles for Targeted Dual-Drug Cancer Therapy**

Murali Mohana Rao Singuru ^a^*, Priyanka Bhattacharyya ^a^, Mingxu You ^a,b,^*

^a^ Department of Chemistry, University of Massachusetts Amherst, 710 N. Pleasant St, Amherst, MA 01003, USA

^b^ Molecular and Cellular Biology Graduate Program, University of Massachusetts Amherst, 710 N. Pleasant St, Amherst, MA 01003, USA

* Corresponding authors

E-mail address: [murali435@gmail.com](mailto:murali435@gmail.com) (S. Murali Mohana Rao), [mingxuyou@umass.edu](mailto:mingxuyou@umass.edu) (M. You)

**Supplementary Figures**


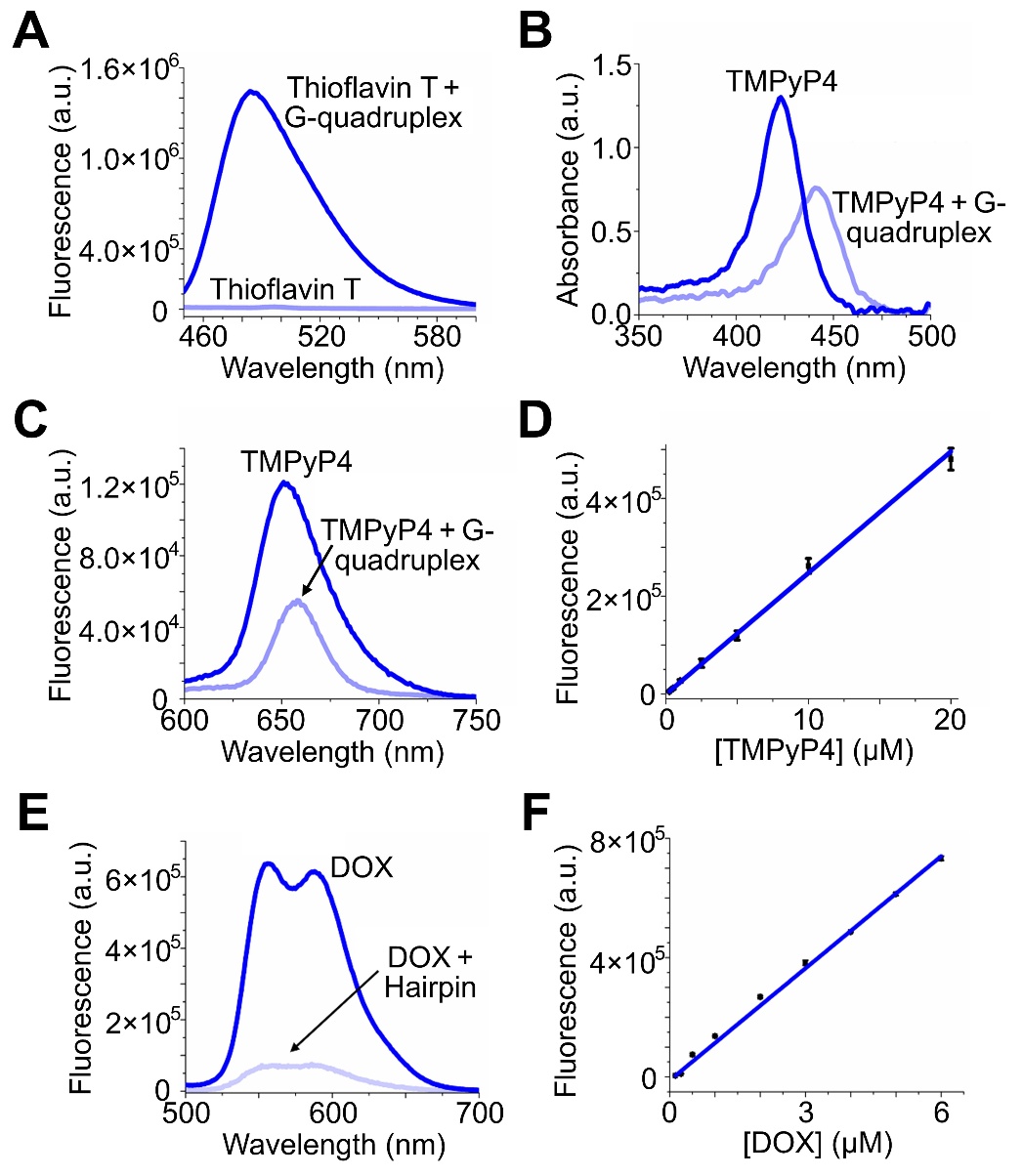


**Figure S1.** Characterization of G-quadruplex/hairpin formation and TMPyP4/DOX binding interactions.

(**A**) Formation of the human telomeric G-quadruplex structure as conformed by thioflavin T (2.0 µM) fluorescence in the presence or absence of 1.0 µM G-quadruplex strand, represented from three independent measurements (n = 2).

(**B**) Representative absorbance spectra (n = 2) of TMPyP4 (5.0 µM) before and after addition of 1.0 µM G-quadruplex DNA.

(**C**) Representative fluorescence spectra (n = 2) of TMPyP4 (5.0 µM) before and after adding 1.0 µM DNA G-quadruplex DNA to assess binding-induced quenching.

(**D**) Fluorescence intensity at 651 nm (excitation at 520 nm) plotted against TMPyP4 concentration, with data shown as mean and standard deviation (SD) values from two independent measurements.

(**E**) Representative fluorescence spectra (n = 2) of DOX (5.0 µM) before and after adding 1.0 µM Hairpin DNA to assess binding-induced quenching.

(**F**) Fluorescence intensity at 581 nm (excitation at 470 nm) plotted against DOX concentration, with data shown as mean and standard deviation (SD) values from two independent measurements.


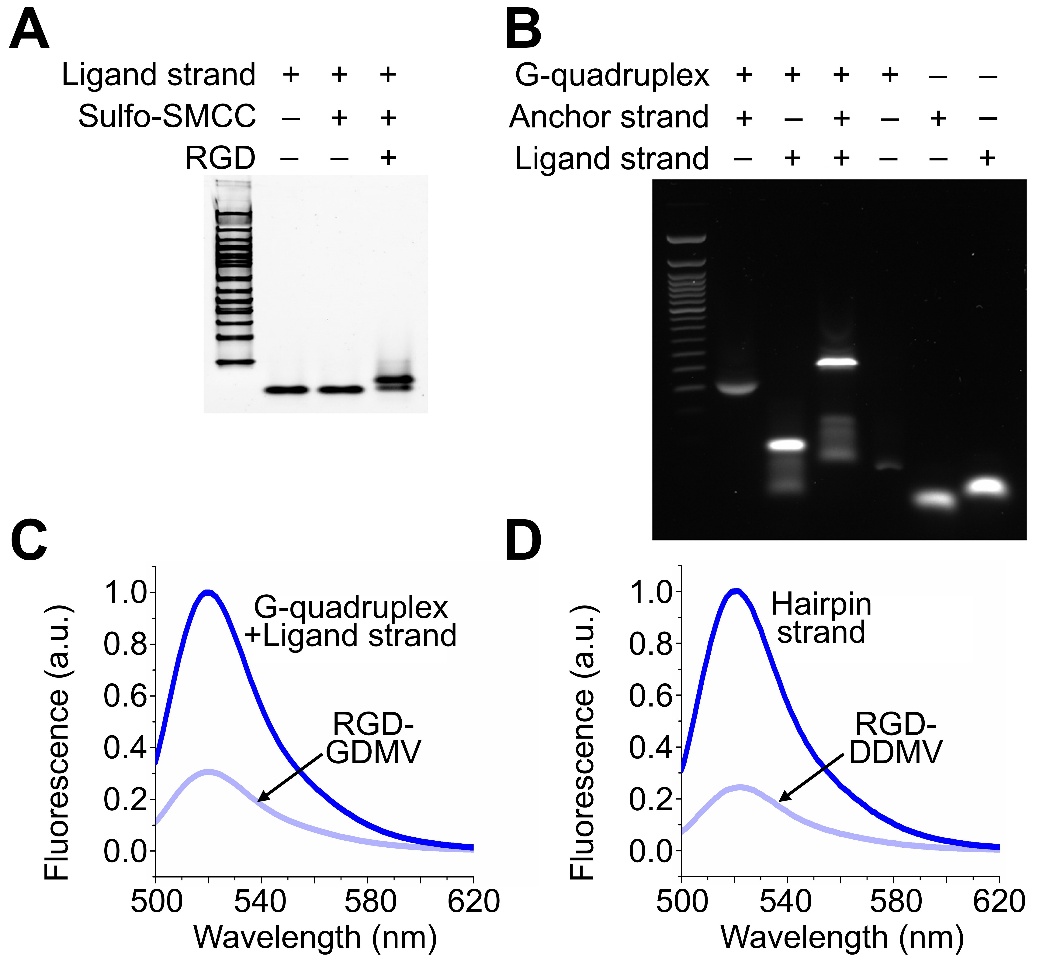


**Figure S2.** Verification and assembly of RGD-modified G-quadruplex DNA nanovehicles.

(**A**) Verification of thiolated DNA ligand strand conjugation with the RGD peptide via sulfo-SMCC using a 12% native polyacrylamide gel (n = 2).

(**B**) Assembly of the G-quadruplex DNA motif, anchor strand, and ligand strand into the RGD-GDMV structure as confirmed by 2% agarose gel electrophoresis (n = 2).

(**C**) Quenching efficiency (n = 2) of preassembled RGD-GDMV determined by comparing its FAM channel fluorescence (dark blue) with a non-quenched control lacking a quencher-labeled anchor strand (light blue).

(**D**) Quenching efficiency (n = 2) of preassembled RGD-DDMV determined by comparing its FAM channel fluorescence (dark blue) with a non-quenched hairpin strand control (light blue).


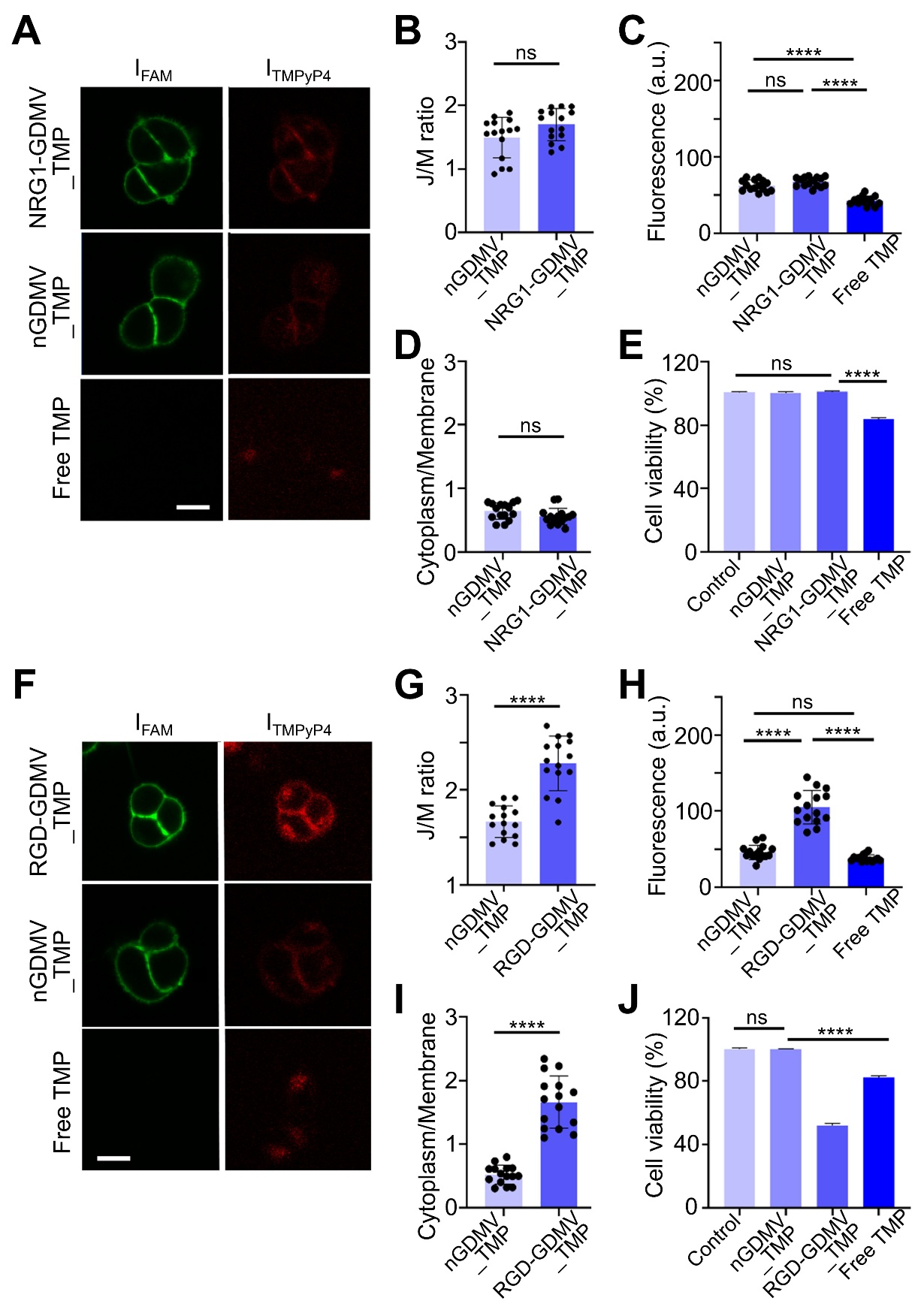


**Figure S3. Validation of tensile force–triggered TMPyP4 release at cell–cell junctions.**

(**A**) Representative confocal images of MCF cells after a 4-hour incubation with 3.0 μM free TMPyP4 or 3 μM TMPyP4-loaded 1.0 μM NRG1-GDMV/nGDMV. Samples were rinsed twice with HEPES before imaging. Scale bar: 20 μm.

(**B**) Quantification of J/M FAM fluorescence ratios (mean ± SD) from 15 junctional cells. Statistical significance was determined by unpaired two-tailed Student’s t-test (ns = not significant).

(**C**) Quantification of cellular TMPyP4 fluorescence (mean ± SD) from at least 15 junctional cells. Significance was assessed by one-way ANOVA (ns = not significant, ****p < 0.0001).

(**D**) Quantitative analysis of cellular cytoplasm over membrane TMPyP4 fluorescence ratios (mean ± SD) measured from 15 junctional cells. Significance was assessed by unpaired two-tailed Student’s t-test (ns = not significant).

(**E**) XTT assay evaluating cytotoxicity after a 4-hour incubation with 3.0 μM free TMPyP4 or 3 μM TMPyP4-loaded 1.0 μM NRG1-GDMV/nGDMV. Untreated cells served as controls. Data represents mean ± SD from three biological replicates. Significance was determined by one-way ANOVA (ns = not significant, ****p < 0.0001).

(**F**) Representative confocal images of MCF cells after a 4-hour incubation with 3.0 μM free TMPyP4 or 3 μM TMPyP4-loaded 1.0 μM RGD-GDMV/nGDMV. Samples were rinsed twice with HEPES before imaging. Scale bar: 20 μm.

(**G**) Quantification of J/M FAM fluorescence ratios (mean ± SD) from 15 junctional cells. Statistical significance was determined by unpaired two-tailed Student’s t-test (****p < 0.0001).

(**H**) Quantification of cellular TMPyP4 fluorescence (mean ± SD) from 15 junctional cells. Significance was assessed by one-way ANOVA (ns = not significant, ****p < 0.0001).

(**I**) Quantification of cellular cytoplasm over membrane TMPyP4 fluorescence ratios (mean ± SD) measured from 15 junctional cells. Significance was assessed by unpaired two-tailed Student’s t-test (****p < 0.0001).

(**J**) XTT assay evaluating cytotoxicity after a 4-hour incubation with 3.0 μM free TMPyP4 or 3 μM TMPyP4-loaded 1.0 μM RGD-GDMV/nGDMV. Untreated cells served as controls. Data represents mean ± SD from three biological replicates. Significance was determined by one-way ANOVA (ns = not significant, ****p < 0.0001).


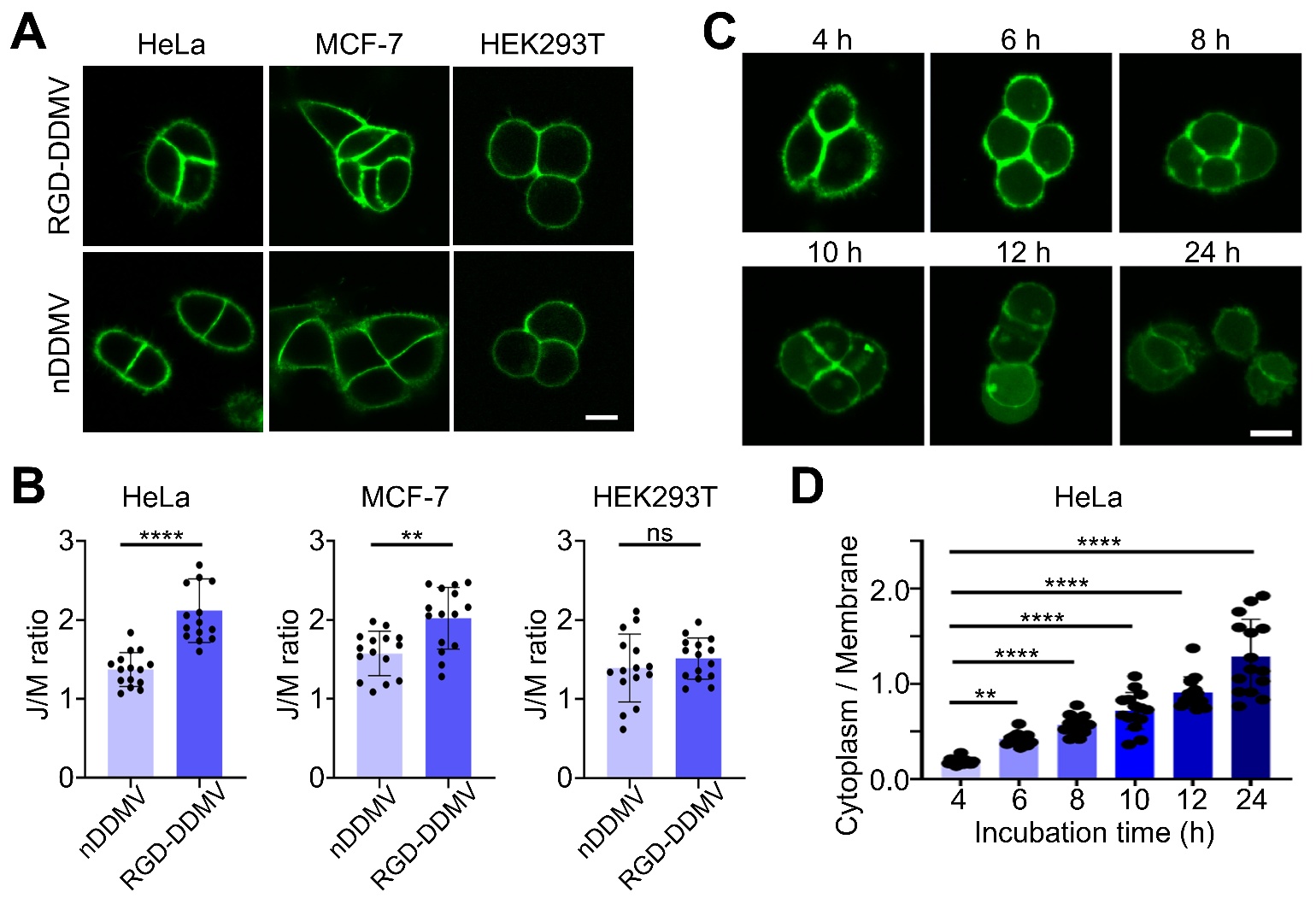


**Figure S4.** **Performance of DDMV in detecting intercellular tensile forces.**

(**A**) Representative fluorescence images (FAM channel) of HeLa, MCF-7, and HEK293T cells after a 30-min incubation with 500 nM RGD-DDMV or nDDMV, followed by two HEPES rinses before imaging. Scale bar: 20 μm.

(**B**) Quantitative analysis of J/M FAM fluorescence ratios (mean ± SD) measured from 15 junctional cells in each case. Statistical significance was evaluated using an unpaired two-tailed Student’s t-test (ns = not significant, **p < 0.01, ****p < 0.0001).

(**C**) Representative fluorescence images (FAM channel) of HeLa cells after 4–24 hours of incubation with 1.0 µM nDDMV. Samples were rinsed twice with HEPES before imaging. Scale bar: 20 μm.

(**D**) Quantitative analysis of cellular cytoplasm over membrane FAM fluorescence ratios (mean ± SD) measured from 15 junctional cells at each time point. It is worth noting that FAM fluorescence may be partially quenched under acidic intracellular conditions in endosomes and lysosomes. Significance was assessed by one-way ANOVA (ns = not significant, **p < 0.01, ****p < 0.0001).


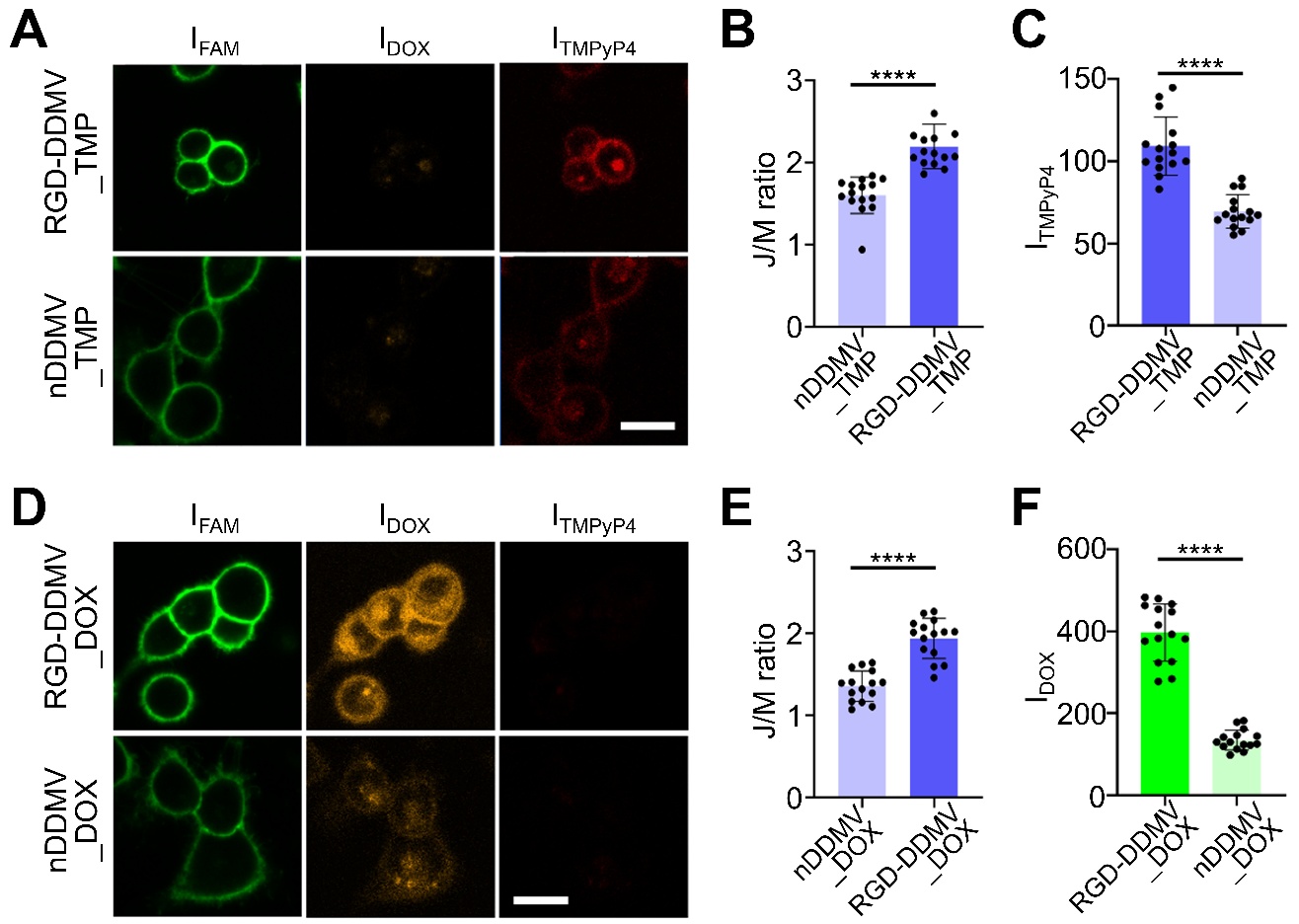


**Figure S5.** Tensile force–driven release of individual drugs at HeLa cell–cell junctions.

(**A**) Representative fluorescence images of HeLa cells after a 4-hour incubation with 3 μM TMPyP4-loaded 1.0 μM RGD-DDMV or nDDMV, followed by two HEPES rinses before imaging. Scale bar: 20 μm.

(**B**) J/M FAM fluorescence ratios quantified (mean ± SD) from 15 junctional cells in each case. Significance was evaluated using an unpaired two-tailed Student’s t-test (****p < 0.0001).

(**C**) Cellular TMPyP4 fluorescence (mean ± SD) measured from 15 junctional cells. Statistical significance was assessed by unpaired two-tailed Student’s t-test (***p < 0.001).

(**D**) Representative fluorescence images of HeLa cells after a 4-hour incubation with 3 μM DOX-loaded 1.0 μM RGD-DDMV or nDDMV, followed by two HEPES rinses before imaging. Scale bar: 20 μm.

(**E**) J/M FAM fluorescence ratios quantified (mean ± SD) from 15 junctional cells after incubation with 3 μM DOX-loaded 1.0 μM RGD-DDMV or nDDMV. Significance was evaluated using an unpaired two-tailed Student’s t-test (****p < 0.0001).

(**F**) Cellular DOX fluorescence (mean ± SD) values measured from 15 junctional cells. Statistical analysis by unpaired two-tailed Student’s t-test (****p < 0.0001).


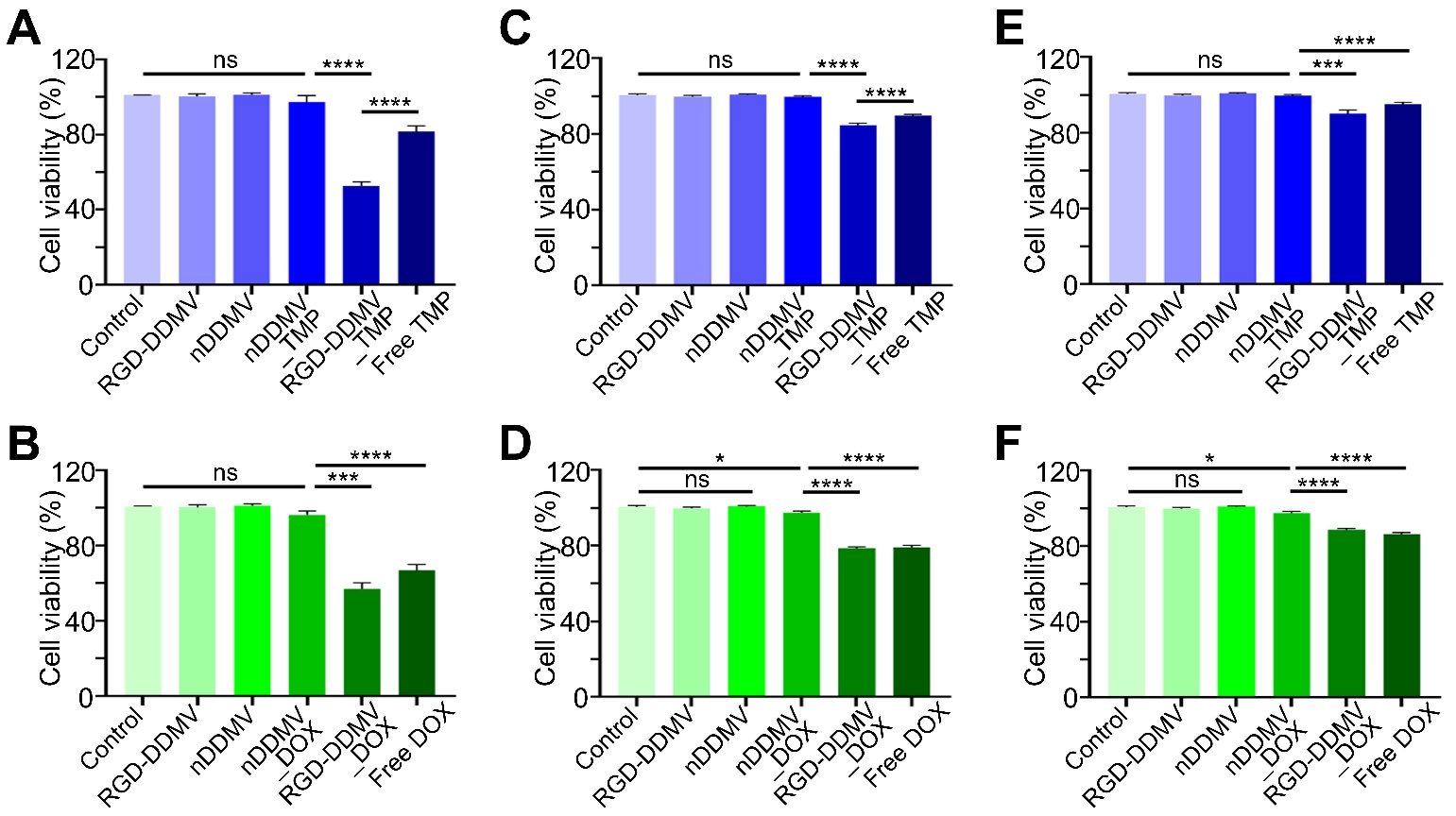


**Figure S6.** Selective anticancer activity of single-drug release and dose-dependent cytotoxicity.

(**A**) HeLa cells were treated for 4 hours with either 3.0 μM free TMPyP4, 3 μM TMPyP4-loaded 1.0 μM RGD-DDMV/nDDMV, or 1.0 μM RGD-DDMV/nDDMV alone. Untreated cells served as controls. Data are shown as mean ± SD from three biological replicates. Statistical analysis: one-way ANOVA (ns = not significant, ****p < 0.0001).

(**B**) Cells treated with 3.0 μM free DOX, 3 μM DOX-loaded 1.0 μM RGD-DDMV/nDDMV, or 1.0 μM RGD-DDMV/nDDMV alone. Results are reported as mean ± SD (n = 3). One-way ANOVA (ns = not significant, ***p < 0.001, ****p < 0.0001).

(**C**) Treatment with 1.5 μM free TMPyP4, ~1.5 μM TMPyP4-loaded 0.5 μM RGD-DDMV/nDDMV, or 0.5 μM RGD-DDMV/nDDMV alone. Mean ± SD (n = 3). One-way ANOVA (ns = not significant, ****p < 0.0001).

(**D**) Treatment with 1.5 μM free DOX, ~1.5 μM DOX-loaded 0.5 μM RGD-DDMV/nDDMV, or 0.5 μM RGD-DDMV/nDDMV alone. Mean ± SD (n = 3). One-way ANOVA (ns = not significant, *p < 0.05, ****p < 0.0001).

(**E**) Treatment with 0.75 μM free TMPyP4, ~0.7 μM TMPyP4-loaded 0.25 μM RGD-DDMV/nDDMV, or 0.25 μM RGD-DDMV/nDDMV alone. Mean ± SD (n = 3). One-way ANOVA (ns = not significant, ***p < 0.001, ****p < 0.0001).

(**F**) Treatment with 0.75 μM free DOX, ~0.7 μM DOX-loaded 0.25 μM RGD-DDMV/nDDMV, or 0.25 μM RGD-DDMV/nDDMV alone. Mean ± SD (n = 3). One-way ANOVA (ns = not significant, *p < 0.05, ****p < 0.0001).


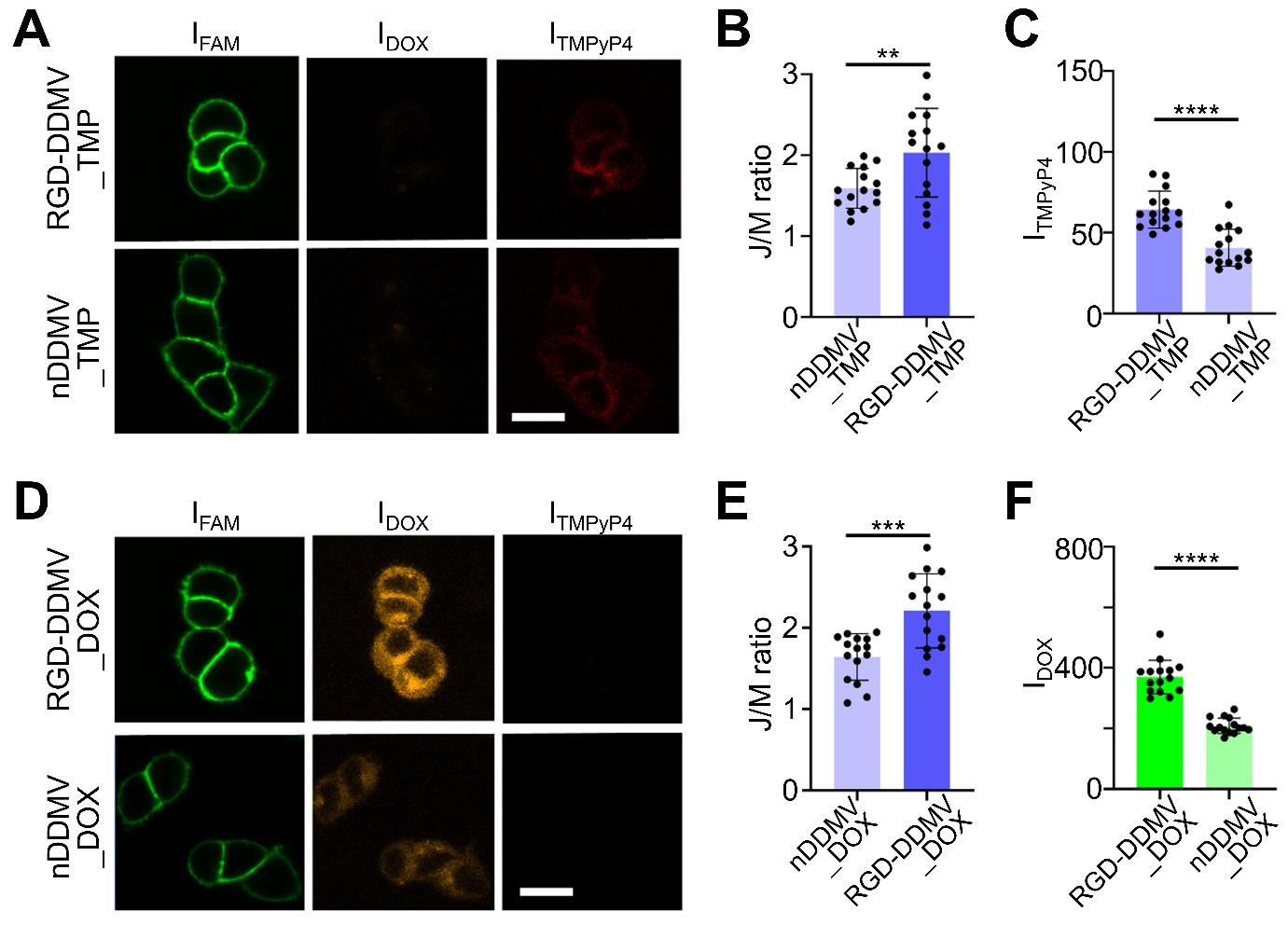


**Figure S7.** Tensile force–driven release of individual drugs at MCF-7 cell–cell junctions.

(**A**) Representative fluorescence images of MCF-7 cells after a 4-hour incubation with 3 μM TMPyP4-loaded 1.0 μM RGD-DDMV or nDDMV, followed by two HEPES rinses before imaging. Scale bar: 20 μm.

(**B**) J/M FAM fluorescence ratios quantified (mean ± SD) from 15 junctional cells in each case. Significance was evaluated using an unpaired two-tailed Student’s t-test (**p < 0.01).

(**C**) Cellular TMPyP4 fluorescence (mean ± SD) measured from 15 junctional MCF-7 cells. Statistical significance was assessed by unpaired two-tailed Student’s t-test (****p < 0.0001).

(**D**) Representative fluorescence images of MCF-7 cells after a 4-hour incubation with 3 μM DOX-loaded 1.0 μM RGD-DDMV or nDDMV, followed by two HEPES rinses before imaging. Scale bar: 20 μm.

(**E**) J/M FAM fluorescence ratios quantified (mean ± SD) from 15 junctional cells after incubation with 3 μM DOX-loaded 1.0 μM RGD-DDMV or nDDMV. Significance was evaluated using an unpaired two-tailed Student’s t-test (***p < 0.001).

(**F**) Cellular DOX fluorescence (mean ± SD) values measured from 15 junctional cells. Statistical analysis by unpaired two-tailed Student’s t-test (****p < 0.0001).


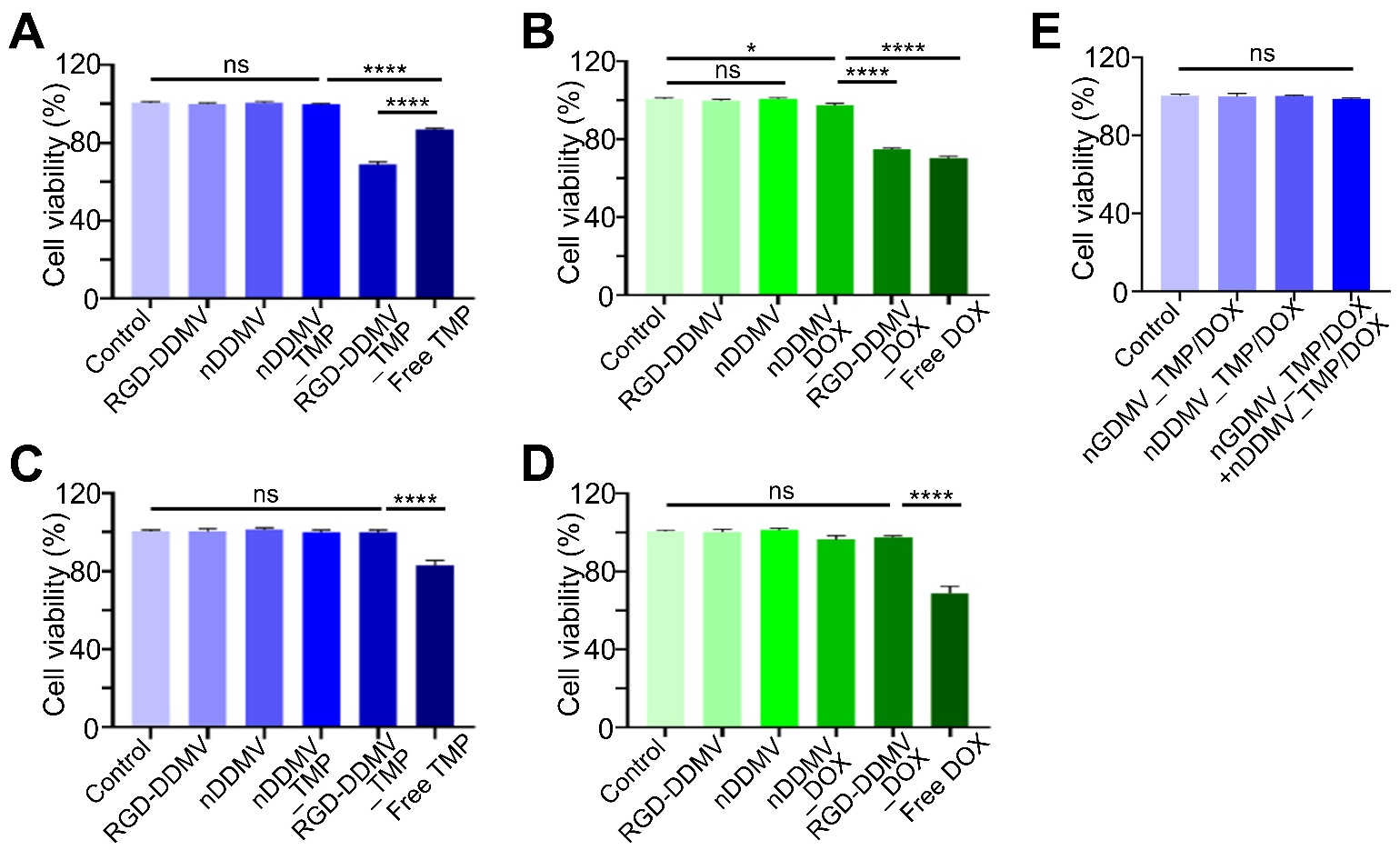


**Figure S8.** Selective single-drug release and cytotoxicity in cancer vs. low-tension cells.

(**A**) MCF-7 cells were treated for 4 hours with either 3.0 μM free TMPyP4, 3 μM TMPyP4-loaded 1.0 μM RGD-DDMV/nDDMV, or 1.0 μM RGD-DDMV/nDDMV alone. Untreated cells served as controls. Data are shown as mean ± SD from three biological replicates. Statistical analysis: one-way ANOVA (ns = not significant, ****p < 0.0001).

(**B**) MCF-7 cells treated with 3.0 μM free DOX, 3 μM DOX-loaded 1.0 μM RGD-DDMV/nDDMV, or 1.0 μM RGD-DDMV/nDDMV alone. Results are reported as mean ± SD (n = 3). One-way ANOVA (ns = not significant, *p < 0.05, ****p < 0.0001).

(**C**) HEK293T cells treated with 3.0 μM free TMPyP4, 3 μM TMPyP4-loaded 1.0 μM RGD-DDMV/nDDMV, or 1.0 μM RGD-DDMV/nDDMV alone. Mean ± SD (n = 3). One-way ANOVA (ns = not significant, ****p < 0.0001).

(**D**) HEK293T cells treated with 3.0 μM free DOX, 3 μM DOX-loaded 1.0 μM RGD-DDMV/nDDMV, or 1.0 μM RGD-DDMV/nDDMV alone. Mean ± SD (n = 3). One-way ANOVA (ns = not significant, ****p < 0.0001).

(**E**) HEK293T cells treated for 4 hours with 3 μM TMPyP4/DOX-loaded 1.0 μM nGDMV and nDDMV, both individually and in combination. Untreated cells served as controls. Results are reported as mean ± SD (n = 3). One-way ANOVA (ns = not significant).

**Supplementary Table**

**Table S1.** Genetic sequences of DNA strands used in this study.

| **Oligo names** | **Sequences (5’ – 3’)** |
| --- | --- |
| Anchor strand | Epoch Eclipse-CGCATCTGTGCGGTATTTCACCCC-Cholesterol |
| Human telomeric DNA (G-quadruplex) | CCCGTGAAATACCGCACAGATGCGTTTAAAGGGTTAGGGTTAGGGTTAGGGAATTTTAAATTTGTTTCTTTCTTGTTT |
| Ligand strand (FAM) | HS-TTTGCTGGGCTACGTGGCGCTCTT-FAM |
| Ligand strand | HS-TTTGCTGGGCTACGTGGCGCTCTT |
| Hairpin strand | AATTGAAAGATCACAGACATCAGTTTGCATGCACGTTTTTTTCGTGCATGCTTTAAACAAGAAAGAAACAAATTT-FAM |
